# A dynamical optimal control theory and cost-effectiveness analyses of the HBV and HIV/AIDS co-infection model

**DOI:** 10.3389/fpubh.2024.1444911

**Published:** 2024-10-29

**Authors:** Shewafera Wondimagegnhu Teklu, Abushet Hayalu Workie

**Affiliations:** Department of Mathematics, Natural and Computational Sciences, Debre Berhan University, Debre Berhan, Ethiopia

**Keywords:** HBV and HIV co-infection, protection, stability analysis, optimal control measure, cost-effective analysis

## Abstract

Studies have shown that the co-infection of Human Immunodeficiency Virus (HIV) and Hepatitis B Virus (HBV) poses a major threat to the public health due to their combined negative impacts on health and increased risk of complications. Even though, some scholars formulated and analyzed the HBV and HIV co-infection model they did not consider the compartment that contains protected individuals against both HBV and HIV infections. They incorporated the optimal control theory and cost-effectiveness analysis simultaneously. With this in mind, we are motivated to formulate and analyze the HBV and HIV co-infection model, considering the protected group and incorporating optimal control theory and cost-effectiveness. In this study, we have theoretically computed all of the models disease-free equilibrium points, all the models effective reproduction numbers and unique endemic equilibrium points. The two sub-models disease-free equilibrium points are locally as well as globally asymptotically stable whenever their associated effective reproduction numbers are less than one. We reformulated the optimal control problem by incorporating five time-dependent control measures and conducted its theoretical analysis by utilizing the Pontryagin's maximum principle. Using the fourth order Runge–Kutta numerical method and MATLAB ODE45, we performed the numerical simulations with various combinations of control efforts to verify the theoretical results and investigate the impacts of the suggested protection and treatment control strategies for both the HBV and HIV diseases. Also, we carried out a cost-effectiveness analysis of the proposed control strategies. Eventually, we compared our model results with other researcher similar model results whenever cost-effectiveness analysis is not carried out the findings of this particular study suggest that implementing each of the proposed control strategies simultaneously has a high potential to reduce and control the spread of HBV and HIV co-infections in the community. According to the cost-effectiveness analysis, implementing the HBV treatment and the HIV and HBV co-infection treatment measures has a high potential effect on reducing and controlling the HBV and HIV co-infection transmission problem in the community.

## Introduction

HBV is a microbial pathogenic virus that greatly influences the normal work of individuals' livers. About two billion individuals throughout the world have been infected with the HBV epidemic among which chronic HBV has affected more than 350 million individuals throughout nations in the world (1–5). Millions of individuals have been died from chronic HBV stages (liver cirrhosis and cancer) and it spreads through direct contact and indirect transmission, such as through blood contact or during birth (1). The two most common stages of HBV disease are acute and chronic hepatitis stages (6). Through the first 180 days after individuals are exposed to the hepatitis B virus, their immune system may be able to remove the HBV virus, resulting in a complete recovery. However, sometimes the HBV infection may progress to the chronic HBV infection stage (6).

AIDS (acquired deficiency syndrome) is a highly infectious disease that is caused by HIV (human immunodeficiency virus). It is one of the major life threatening and most destructive epidemic diseases in history (9). It has affected approximately 70 million individuals across various nations worldwide (7). According to the Joint United Nations Programme on HIV/AIDS (UNAIDS) report, retroviruses are spreading throughout the world (7–9). Since 1981, it has been declared as a global pandemic. In 2016, 36.7 million individuals were living with HIV/AIDS, and more than two thirds of those individuals in the world are living in sub-Saharan African countries (8, 10). The most common infection stages of HIV are acute, dormancy, and AIDS (11). HIV/AIDS can be transmitted from an infected individual to a healthy individual through direct or indirect transmission, and its possible control measures are preventive measures and treatment regimens (10).

A co-infection is the co-occurrence of two or more pathogens (infections) on a single individual at the population level (8). HIV and HBV are the most common viral infectious diseases and share similar modes of transmission. The HBV and HIV co-infection disease is a common infectious disease throughout the world (2, 12, 13). More than 10% of individuals infected with HIV have been reported as chronically infected with HBV, and the HBV-HIV co-infection highly increases the risk for liver related morbidity and mortality as compared with the HIV mono-infection (1, 14, 15). HIV/AIDS infection continues to be one of the most common public health problems, with additional risk of HCV and/or HBV co-infection (3, 12, 16).

Mathematical modeling is the process of representing real-world situations using mathematical terms and expressions. It plays a vital role to understand and predict the future behaviors and results for the real-world problem solutions (49). By combining the mathematical techniques with biological and epidemiological knowledge, researchers are able to simulate different scenarios, search for different control measures and different interventions, and conduct a public health decision-making process (49). From the diverse branches of mathematical modeling one can motivate one to study about eco-epidemiological, ecological, and epidemiological modeling. Epidemiological modeling is the study of the infectious disease transmission dynamics at the population level. It plays a crucial role in the study of transmission dynamics such as HBV and HIV infectious diseases (17). There are different single-infection disease studies using the integer-order derivative approaches (18–22) and using fractional order derivative approaches (23–30). On the other hand, the interactions between two infectious diseases, particularly the HBV and HIV, have a negative impact on the community and have become a global concern these days. Therefore, different researchers have given attention to studying the spreading dynamics of HBV and HIV co-infection within the community (2, 31, 32).

Different researchers have studied different infectious diseases with mathematical modeling approaches; for instance, Jan et al. (23) formulated and analyzed the dynamical behavior and chaotic phenomena of HIV infection through fractional order derivatives primarily with the Atangana–Baleanu derivative in the Caputo sense, to investigate the dynamics of CD4+T-cells in HIV infection. Bowong et al. (2) formulated and presented the HBV and HIV co-infection deterministic model, and they carried out numerical simulations for the full co-infected model to verify the analytical results. Endashaw and Mekonnen (32) investigated the impact of HBV vaccination and HBV and HIV treatments on the spreading dynamics of HBV and HIV/AIDS co-infection. Their findings revealed that implementing HBV vaccination, HBV, HIV/AIDS, and HBV and HIV/AIDS co-infection treatments at the highest possible rate is recommended to control the transmission of HBV and HIV/AIDS co-infection within the community. Endashaw et al. (31) modified the HBV and HIV/AIDS co-infection model (32) by incorporating vertical transmission, i.e., transmission from mother to child, and medical interventions. According to numerical simulations, increasing the HBV and HIV mother-to-child vertical transmission rates exacerbated the HBV and HIV/AIDS co-infection. Based on the findings of the studies conducted by other researchers, we are motivated to address the gap by formulating the HBV and HIV co-infection model incorporating optimal control theory and cost-effectiveness investigation. Ullah et al. (51) developed and analyzed a new HBV and HIV co-infection model with vaccination and asymptomatic transmission using real data collected from Taiwan. The study considered vaccination, exposed and asymptomatic compartments and from the numerical simulation results, we observed that the vaccine and fractional parameters changed the proposed model state variables, as well as how the solutions behaved and how quickly they reached the model's equilibrium. A maximum vaccination effort against HBV has a great effect on the HIV and HBV co-infection spreading in the community. Yusuf and Idisi (52) formulated and analyzed the HIV and HBV co-epidemics spreading dynamics. Nampala et al. (53), formulated and analyzed modeling and investigating hepatotoxicity and antiretroviral therapeutic effect in HIV/HBV co-infection spreading dynamics. However, all of the above HBV and HIV co-infection models do not incorporate the protected group against both infections (by education or condom use) and optimal control theory and cost-effectiveness analysis into the transmission dynamics of these diseases.

Optimal control and cost-effectiveness analyses are vital tools to investigate the possible impacts of intervention strategies against infectious disease-spreading dynamics. It provides public stakeholders and policymakers with the right decision on which possible control intervention measure has the most beneficial economic value (and is less expensive). For instance, Awoke and Semu (33) formulated a TB and HIV co-infection model with optimal control theory in the presence of behavior modification. They investigated the optimal impacts of their proposed control strategies, and from their cost-effectiveness analysis results they found that the treatment control measure is more effective than the preventive control strategies. Shang (54) formulated a mathematical model to investigate the impacts of optimal control strategies for virus spreading in inhomogeneous epidemic dynamics. The study investigated the spread of virus/worm in computer networks with a view to addressing cyber security problems using the same approaches used in epidemic models. Ahmed et al. (55) investigated the optimal treatment strategies to control acute HIV infection. From our findings, we found that early initiation of treatment has a profound impact on both improving the quality of life and reducing the economic costs of therapy. Kamrujjaman et al. (56) investigated the dynamics of a diffusive vaccination model with therapeutic impact and non-linear incidence in the field of epidemiology. Ahmed et al. (57) investigated the dynamics of a viral infection under treatment. Asamoah et al. (46) investigated the global stability and cost-effectiveness analysis on the spread of coronavirus disease 2019 (COVID-19) spreading by considering the impact of the environment using authentic data from Ghana. Teklu (44) investigated the impacts of optimal control strategies on the HBV and COVID-19 co-infection transmission dynamics. Kotola et al. (37) formulated and analyzed a mathematical model for the HIV/AIDS and COVID-19 co-infection with bifurcation and optimal control analysis. Khondaker et al. (58) formulated and analyzed the COVID-19 transmission model with optimal control theory, physical distance, and treatment.

Therefore, it should be mentioned that researcher studies like (2, 31, 32, 51–53) formulated and analyzed the HBV and HIV co-infection models by considering HBV vaccination or/and treatments for both infections. However, these models do not incorporate the protected group against both infections (through education or condom use) and optimal control theory and cost-effectiveness analysis in the transmission dynamics of these diseases. Our study assesses the impact of protection strategies for both HBV and HIV infections as well as treatments for both infections on the control and management of the HBV and HIV co-infection spreading through a mathematical modeling approach that incorporates optimal control theory and cost-effective analysis. To the best of the our understanding from literature review the HBV and HIV co-infection model, comprising seven mutually exclusive compartments, such as being susceptible, protected against both HBV and HIV infections, infected solely with HBV, infected solely with HIV, co-infected with HVB and HIV, treated from HBV infection, and treated from HIV infection or/and HBV and HIV co-infection, with optimal control theory and cost-effectiveness analysis, is being considered for the first time. Furthemore, we performed a detailed theoretical and quantitative analysis of the formulated HBV and HIV co-infection model. It is along the same lines of idea as Endashaw et al. (31) and Endashaw and Mekonnen (32), however the approach is very different and we use optimal control theory and the cost-effectiveness approach. From our literature review understanding we have verified that there is no HBV and HIV co-infection compartmental model that considers a protected compartment, optimal control theory, and cost-effectiveness analysis. To this end, our study considers individuals who are protected against both HBV and HIV infections and formulates the HBV and HIV co-infection compartmental model with optimal control theory and a cost-effectiveness approach to obtain a better understanding of the spreading dynamics and control mechanisms of the HBV and HIV diseases. The main contributions of this study can be organized as follows: a new HBV and HIV co-infection model that contains the protected group and describes the co-dynamics characteristics of HBV and HIV with optimal control theory by applying the Pontryagin's Maximum Principle is formulated. A detailed theoretical analysis of the proposed co-infection model is presented, and a cost-effectiveness analysis using the well-known incremental cost-effectiveness ratio (ICER) is performed. This results of this particular study suggest the potential of the proposed control strategies used to implement for reducing and controlling the HIV and HBV co-infection disease.

### Research gap and significance of the present study

From our literature review part, we confirmed that no mathematical model researchers of HIV and HBV co-infection disease considered the number of people who are protected against both HIV and HBV infections using education and condom, optimal control theory, and cost-effectiveness analysis in the dynamics of this co-infection disease, despite the fact that some researchers formulated and analyzed the HIV and HBV co-infection dynamics. In view of this, we are motivated to develop and evaluate the HIV and HBV co-infection spreading dynamical system in this study, which includes five time-dependent control measures with 31 possible combinations of these control strategies and their cost-effectiveness analysis. The primary objective is to investigate the most economical and ideal control approach. The HIV and HBV co-infection spreading model explained the co-existing characteristics of both infections through time-dependent control strategies. A comprehensive qualitative (mathematical) analysis of the HIV and HBV co-infection spreading dynamical system is presented. The Incremental Cost-Effectiveness Ratio (ICER) approach is used to perform cost-effectiveness analyses. Graphical representations of the suggested control strategies combined in various scenarios are presented, and the results are compared. These constitute the most significant contributions of this study.

The main objective of this study is to find an optimal trajectory for the proposed control strategies that minimizes both the number of co-infected individuals and costs by formulating and analyzing the HBV and HIV co-infection models with optimal control theory. Without carrying out a cost-effectiveness analysis of the optimal control problem, the findings of this particular study suggested that the implementation of each of the proposed controlling strategies simultaneously has a great potential to reduce and control the HBV and HIV co-infection spreading in the community, but cost-effectiveness analysis investigated that Strategy 15 [i.e., implementing HBV treatment and the HIV and HBV co-infection treatment measures (*c*_4_≠0, *c*_5_≠0, *c*_1_ = *c*_2_ = *c*_3_ = 0) simultaneously] has a high potential to reduce and control the HIV and HBV co-infection spreading in the community among each of the 31 proposed control strategies under consideration in the study.

## Methods

In this section, to formulate our proposed HBV and HIV co-infection model, we subdivide the total number of human population at a given time, t denoted by *N*(*t*), into seven mutually exclusive categories based on their infection status. These categories include the number of people who are susceptible to either HBV or HIV, denoted by [*S*(*t*)], the number of people who are protected against both HBV and HIV infections, denoted by [*P*(*t*)], the number of people who are infected solely with HBV, denoted by [*I*_*B*_(*t*))], the number of people who are infected only with HIV, denoted by [*I*_*H*_(*t*)], the number of people who are co-infected with HVB and HIV, denoted by [*C*(*t*)], the number of people who are treated from HBV infection, denoted by [*T*_*B*_(*t*)], and the number of people who are treated from HIV infection or/and HBV and HIV co-infection, denoted by [*T*(*t*)], such that


(1) $$\begin{array}{l} N\left(t\right)=\;S\left(t\right)+P\left(t\right)+I_{B}\left(t\right)+I_{H}\left(t\right)+C\left(t\right)+T_{B}\left(t\right)+T\left(t\right). \end{array}$$

The following are the assumptions of the proposed model

- Individuals in each category are homogeneously mixing,
- Treated individuals do not transmit the diseases due to awareness,
- Individuals in each category are subject to natural mortality,
- The human population is not constant,
- There is no simultaneous dual-infection transmission,
- There is no vertical transmission of HBV infection,
- HIV vertical transmission has been considered,
- There is no permanent HBV infection,
- The protection may not be 100% effective,
- The co-infected individuals (*C*(*t*)) are more infectious than single infected individuals (*I*_*B*_(*t*)) and (*I*_*H*_(*t*)); therefore, the constants 1 ≤ ϑ_1_ < ∞, and 1 ≤ ϑ_2_ < ∞ are the modification parameters used to compare the degree of infectiousness of the co-infected individuals with HBV and HIV single-infection individuals, respectively.
- Individuals who are susceptible to either HBV or HIV infection acquire HBV or HIV infection at the force of infection rates described respectively by:

(2) $$\begin{array}{l} \lambda_{B}\left(t\right)=\frac{\beta_{1}}{N\left(t\right)}\left(I_{B}\left(t\right)+\vartheta_{1}C\left(t\right)\right)\;, \end{array}$$

(3) $$\begin{array}{l} \lambda_{H}\left(t\right)=\frac{\beta_{2}}{N\left(t\right)}\left(I_{H}\left(t\right)+\vartheta_{2}C\left(t\right)\right)\;, \end{array}$$

where the parameters described by β_1_, and, β_2_ are the HBV and HIV transmission rates respectively and *N*(*t*) is the total population stated in Equation 1.

**Figure 1:**
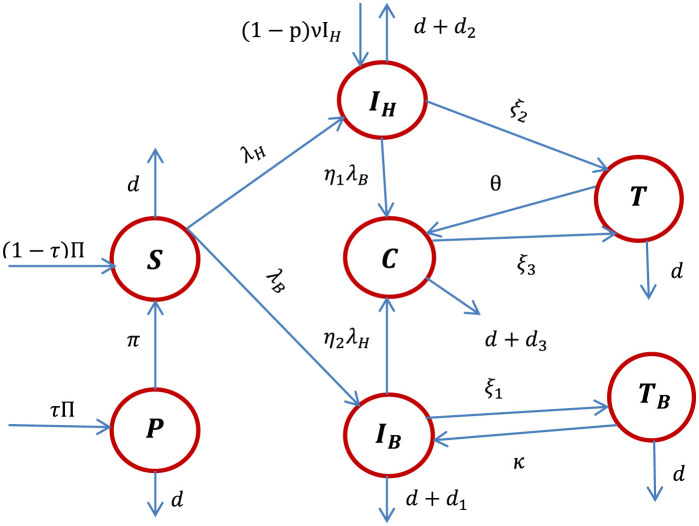
The schematic diagram of the HBV and HIV co-infection dynamics where λ_*H*_ and λ_*B*_ are stated in Equations 1, 2, respectively.

Using the model state variable definitions, the model assumptions described above, and the descriptions of parameters stated in Table 1, the HBV and HIV co-infection transmission dynamics schematic diagram is shown in Figure 1.

**Table 1:** Description of parameters used to formulate and simulate the co-infection model.

**Symbols**	**Biological interpretation**	**Values**	**References**
*d*	Human natural mortality rate	0.01	(11)
Π	Human recruitment rate	250	(34)
τ	Portion of HBV or/and HIV protection	0.006	(35)
π	Protection loss rate	0.59	(36)
η_1_	Parameter that shows an HIV-infected individual is more risky than a susceptible individual for HBV infection	1.2	Assumed
η_2_	Parameter that shows an HBV-infected individual is more risky than a susceptible individual for HIV infection	1.1	Assumed
*d*_1_	HBV infection death rate	0.1	(31)
*d*_2_	HIV infection death rate	0.333	(7)
*d*_3_	HBV and HIV co-infection death rate	0.01	(32)
κ	HBV re-infection rate of HBV-treated individuals	0.2	Assumed
θ	HBV re-infection rate of co-infected treated individuals	0.3	Assumed
β_1_	HBV infection transmission rate	0.3425	(37)
β_2_	HIV infection transmission rate	0.04	(31)
ξ_1_	HBV infection treatment rate	0.3	(31)
ξ_2_	HIV infection treatment rate	0.3	(32)
ξ_3_	HBV and HIV co-infected treatment rate	0.015	(32)
*p*	Probability of death of newborns infected with HIV at birth	0.2	(31)
ν	Vertical transmission rate of HIV from mother to child at birth	0.3	(31)

Using the schematic diagram shown in Figure 1, the HBV and HIV co-infection dynamical systems are represented by the systems of differential equations given by:


(4) $$\begin{array}{lll} \frac{dS}{dt} & = & \left(1-\tau \right)\text{Π}+\pi P-\left(\lambda_{H}+\lambda_{B}+d\right)S,\\ \frac{dP}{dt} & = & \tau \text{Π}-\left(\pi +d\right)P,\\ \frac{dI_{H}}{dt} & = & \lambda_{H}S+\left(1-p\right)\text{ϕ}\nu I_{H}-\left(\eta_{1}\lambda_{B}+\xi_{2}+d_{2}+d\right)I_{H},\\ \frac{dI_{B}}{dt} & = & \lambda_{B}S+\kappa T_{B}-\left(\eta_{2}\lambda_{H}+\xi_{1}+d_{1}+d\right)I_{B},\\ \frac{dC}{dt} & = & \eta_{1}\lambda_{B}I_{H}+\eta_{2}\lambda_{H}I_{B}+\text{θ}T-\left(\;\xi_{3}+d_{3}+d\right)C,\\ \frac{dT}{dt} & = & \xi_{2}I_{H}+\xi_{3}C-\left(\theta +d\right)T,\\ \frac{dT_{B}}{dt} & = & \xi_{1}I_{B}-\left(\kappa +d\right)T_{B}, \end{array}$$

with initial population quantified by *S*(0) = *S*^0^ ≥ 0, *P*(0) = *P*^0^ ≥ 0, $I_{H}\left(0\right)=I_{H}^{0}\geq 0$, $I_{B}\left(0\right)=I_{B}^{0}\geq 0$, *C*(0) = *C*^0^ ≥ 0, *T*^0^ = *T*^0^ ≥ 0, and $T_{B}\left(0\right)=T_{B}^{0}\geq 0$.

### Non-negativity and boundedness of the co-infection model solutions

In this subsection, we examine the fundamental outcomes pertaining to the solutions of the co-infection dynamical system (4), which hold significant importance in both mathematical and epidemiological interpretations. Each of the state variables included in the co-infection model (4) considers the human population; therefore, it is necessary to reveal that all the state variables are non-negative and bounded.

**Theorem 1:** The solutions to the HBV and HIV co-infection model (4) are non-negative, unique, and bound in the region represented by:


(5) $$\begin{array}{l} \text{Ω}=\left\{\left(S,P,\;I_{H},\;I_{B},C,T,T_{B}\right)\epsilon \;{\mathbb{R}}_{+}^{7}:0\leq N\leq \frac{\text{Π}}{d}\right\}. \end{array}$$

**Proof:** All functions described in the right-hand side of the co-infection model (4) are *C*^1^ on ${\mathbb{R}}_{+}^{7}.$ According to the Picard–Lindelöf theorem the co-infection model (4) has a unique solution. Let the dynamical system (4) be written as *y*′ = *g*(*y, t*) where *y* = (*S, P, I*_*H*_, *I*_*B*_, *C, T, T*_*B*_) and *g* is the right-hand side of the model (4). According to the results of Picard–Lindelöf theorem the function *g*(*y, t*) has the property of


(6) $$\begin{array}{l} f_{i}\left(S,P,\;I_{H},\;I_{B},C,T,T_{B}\right)\geq \;0. \end{array}$$

where *y* = (*S, P, I*_*H*_, *I*_*B*_, *C, T, T*_*B*_) stated in (6) is *y* ∈ [0, ∞]^7^. Since there exists a unique solution for the co-infection model (4), it follows that *y*(*t*) ∈ [0, ∞]^7^ for all *t* ≥ 0, whenever *y*(0) ≥ 0. The rate of change of total human population derived by $\frac{dN}{dt}=\frac{dS}{dt}+\frac{dP}{dt}+\frac{dI_{H}}{dt}+\frac{dI_{B}}{dt}+\frac{dC}{dt}+\frac{dT_{B}}{dt}+\frac{dT}{dt}\;$is governed by;


(7) $$\begin{array}{l} \frac{dN}{dt}=\text{Π}-dN-d_{2}I_{H}-d_{1}I_{B}-d_{3}C+\left(1-p\right)\nu I_{H}\leq \text{Π}-dN. \end{array}$$

Solving Equation 7, we have derived the solution for *N*(*t*) given by $N\left(t\right)\leq {N_{0}e}^{-dt}+\frac{\text{Π}}{d}\left(1-e^{-dt}\right)$. Therefore, for the initial population illustrated in the co-infection model (4) with the property 0 ≤ *N*_0_, we have determined the result $0\leq N\left(t\right)\leq \frac{\text{Π}}{d}$ which implies that the solution of the HBV and HIV co-infection model (4) exists, is unique, and is bound in a feasible region Ω given in Equation 5.

Consequently, the dynamics of the co-infection dynamical system (4) preserve the non-negativity of the states, as demonstrated in previous studies (34, 36, 37, 44). Thus, it is evident that the co-infection model (4) is positively invariant in the region $\text{Ω}=\left\{\left(S,P,\;I_{H},\;I_{B},C,T,T_{B}\right)\epsilon \;{\mathbb{R}}_{+}^{7}:0\leq N\leq \frac{\text{Π}}{d}\;\right\}$.

**Remark 1:** The region $\text{Ω}=\left\{\left(S,P,\;I_{H},\;I_{B},C,T,T_{B}\right)\epsilon \;{\mathbb{R}}_{+}^{7}:0\leq N\leq \frac{\text{Π}}{d}\right\}$ is invariant and attracting for the co-infection dynamical system (4). Thus, the co-infection model (4) is both mathematically and epidemiologically well-posed, and it is sufficient to consider the dynamics of the flow generated by the dynamical system (4) in Ω.

### Disease-free equilibrium point and its stability

The complete co-infection dynamical system (4) disease-free equilibrium point $\left(E_{BH}^{0}\right)$ is determined by making all the right-hand side equations equal to zero, and assuming that there is no disease in communities (i.e., *I*_*B*_ = *I*_*H*_ = *C* = *T*_*B*_ = *T* = 0). Thus, the disease-free equilibrium point of the dynamical system described in Equation 4 is given by:


(8) $$\begin{array}{l} E_{BH}^{0}=\left(S^{0},P^{0},I_{B}^{0},I_{H}^{0},C^{0},T_{B}^{0},\;T^{0}\right)\\ \;=\left(\frac{\pi \text{Π}+d\text{Π}\left(1-\tau \right)}{d\left(\pi +d\right)},\frac{\tau \text{Π}}{\pi +d},0,0,0,0,0\right). \end{array}$$

Using the same procedures stated in (38), we have determined the expressions


$$\begin{array}{r} \begin{array}{c} \;\\ F \end{array}i\left(x\right)=\left[\begin{array}{c} \lambda_{H}S+(1-p)\nu I_{H}\\ \lambda_{B}S\\ 0\\ 0 \end{array}\right],\\ \text{and }V_{i}\left(x\right)=\left[\begin{array}{c} \left(\eta_{1}\lambda_{B}+\xi_{2}+d_{2}+d\right)I_{H}\\ \left(\eta_{2}\lambda_{H}+\xi_{1}+d_{1}+d\right)I_{B}-\kappa T\\ \left(\;\xi_{3}+d_{3}+d\right)C-\eta_{1}\lambda_{B}I_{H}-\eta_{2}\lambda_{H}I_{B}-\theta T\\ \left(\theta +d\right)T-\xi_{2}I_{H}-\xi_{3}C \end{array}\right]. \end{array}$$

Since the reproduction number is computed at the HBV and HIV co-infection disease-free equilibrium point given by $E_{BH}^{0}\;$ stated in Equation 8, such that *N*^0^ = *S*^0^+*P*^0^ and making the infected compartments zero as *I*_*H*_ = *I*_*B*_ = *C* = *T*_*B*_ = *T* = 0, finally, we have computed the results given by:


$$\begin{array}{c} F=\left[\begin{array}{cccc} \frac{\beta_{2}S^{0}}{S^{0}+P^{0}}+\left(1-p\right)\nu & 0 & \frac{\beta_{2}\vartheta_{2}S^{0}}{S^{0}+P^{0}} & 0\\ 0 & \frac{\beta_{1}S^{0}}{S^{0}+P^{0}} & \frac{\beta_{1}\vartheta_{1}S^{0}}{S^{0}+P^{0}} & 0\\ 0 & 0 & 0 & 0\\ 0 & 0 & 0 & 0 \end{array}\right],\\ V=\left[\begin{array}{cccc} \left(\xi_{2}+d_{2}+d\right) & 0 & 0 & 0\\ 0 & \left(\xi_{1}+d_{1}+d\right) & 0 & 0\\ 0 & 0 & \left(\;\xi_{3}+d_{3}+d\right) & -\theta \\ -\xi_{2} & 0 & -\xi_{3} & \left(\theta +d\right) \end{array}\right], \end{array}$$

and the next-generation matrix given by:


$$\begin{array}{l} FV^{-1}=\left(\begin{array}{cccc} \frac{\beta_{2}\left(\pi +d\left(1-\tau \right)\right)+\left(1-p\right)\nu \left(\pi +d\right)}{\left(\pi +d\right)\left(\xi_{2}+d_{2}+d\right)} & 0 & 0 & 0\\ 0 & \frac{\beta_{1}\pi +\beta_{1}d\left(1-\tau \right)}{\left(\pi +d\right)\left(\xi_{1}+d_{1}+d\right)} & 0 & 0\\ 0 & 0 & 0 & 0\\ 0 & 0 & 0 & 0\\ \; \end{array}\right). \end{array}$$

Therefore, the corresponding eigenvalues of the next-generation matrix *FV*^−1^ are given by:


$$\begin{array}{l} \left\{0,0,\frac{\beta_{2}\left(\pi +d\left(1-\tau \right)\right)+\left(1-p\right)\nu \left(\pi +d\right)}{\left(\pi +d\right)\left(\xi_{2}+d_{2}+d\right)},\frac{\beta_{1}\pi +\beta_{1}d\left(1-\tau \right)}{\left(\pi +d\right)\left(\xi_{1}+d_{1}+d\right)}\;\right\}. \end{array}$$

Thus, the effective reproduction number of the co-infection model is given by


$$\begin{array}{l} R_{HB}=\max\left\{\;\frac{\beta_{2}\left(\pi +d\left(1-\tau \right)\right)+\left(1-p\right)\nu \left(\pi +d\right)}{\left(\pi +d\right)\left(\xi_{2}+d_{2}+d\right)},\;\frac{\beta_{1}\pi +\beta_{1}d\left(1-\tau \right)}{\left(\pi +d\right)\left(\xi_{1}+d_{1}+d\right)}\;\right\}, \end{array}$$

where

$R_{B}=\frac{\beta_{1}\pi +\beta_{1}d\left(1-\tau \right)}{\left(\pi +d\right)\left(\xi_{1}+d_{1}+d\right)}$ is the HBV only infection effective reproduction number and $R_{H}=\frac{\beta_{2}\left(\pi +d\left(1-\tau \right)\right)+\left(1-p\right)\nu \left(\pi +d\right)}{\left(\pi +d\right)\left(\xi_{2}+d_{2}+d\right)}$ is the HIV sub-model effective reproduction number.

**Figure 2:**
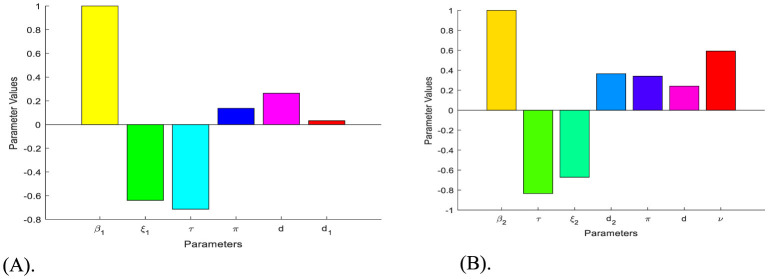
Diagrams that reveal the sensitivity indices of the model parameters with respect to effective reproduction numbers. **(A)** Diagram of the sensitivity indices of the model parameters with respect to${\;R}_{B}$. **(B)** Diagram of the sensitivity indices of the model parameters with respect to${\;R}_{H}$.

**Remark:** Whenever${\;R}_{HB}<1$, the HBV and HIV co-infection spreading will be eliminated in the near future without implementing further control efforts, but if ${\;R}_{HB}>1$, the HBV and HIV co-infection spreads in the community. In order to reduce the HBV and HIV co-infection model effective reproduction number${\;R}_{HB}$, we can vary the model parameters incorporated in${\;R}_{HB}$. Since ${\;R}_{HB}$ is dependent on the model parameters *β*_1_, π, *d*, τ, ξ_1_, *d*_1_, *β*_2_, *p*, ν, ξ_2_, and *d*_2_. From the sensitivity indices results illustrated in the sensitivity analysis section and the sensitivity indices diagram shown in Figure 2, the co-infection model effective reproduction number is directly proportional to some of the model parameters, such as *β*_1_, π, *d*, *β*_2_, *and ν* and also inversely proportional to some of the parameters, like τ, ξ_1_,*d*_1_, ξ_2_, and *d*_2_.

**Theorem 2:** The HBV and HIV co-infection model (4) disease-free equilibrium point $E_{HB}^{0}$ is locally asymptotically stable if $R_{HB}<1$ and is unstable if${\;R}_{HB}>\;1$.

**Proof*:*** The Jacobian matrix $\;J\left(E_{HB}^{0}\right)$ of the HBV and HIV co-infection model (4) at the co-infection disease-free equilibrium point $E_{HB}^{0}\;$is computed by:


$$\begin{array}{l} J\left(E_{HB}^{0}\right)=\left[\begin{array}{ccccccc} -d & \nu & -A\; & -BS^{0} & A\xi_{1}-B\xi_{2} & -A & 0\\ 0 & -C & 0 & 0 & 0 & 0 & 0\\ 0 & 0 & D & 0 & 0 & 0 & 0\\ 0 & 0 & 0 & -E & 0 & 0 & \sigma \\ 0 & 0 & 0 & 0 & -F & \rho & 0\\ 0 & 0 & \xi_{2} & 0 & \xi_{3} & -G & 0\\ 0 & 0 & 0 & \xi_{1} & 0 & 0 & -H\\ \; \end{array}\right], \end{array}$$

where, A$\;=\;\frac{\beta_{2}}{N}S^{0}$, B$\;=\;\frac{\beta_{1}}{N}S^{0}$, C = (π + *d*), D = (1 − τ)ν − (ξ_2_ + *d*_2_ + *d*), E = (ξ_1_ + *d*_1_ + *d*), F = (ξ_3_ + *d*_3_ + *d*), G = (θ + *d*), H = (κ + *d* ).

Then characteristic equation of $J\left(E_{HB}^{0}\right)$ at co-infection model (4) disease-free equilibrium point is given by


$$\begin{array}{l} \left(-C-\lambda \right)\left(D-\lambda \right)\left(-\lambda +d-\right)\\ \left[\left(-E-\lambda \right)\left(-H-\lambda \right)\left(G+\lambda^{2}-FG+F\lambda +\theta \xi_{3}\right)\left(1-\tau \xi_{1}\right)\right]\;=0. \end{array}$$

Then, the solutions of corresponding characteristic equation are


$$\begin{array}{l} \lambda =D,\;\lambda =-\left(d+\pi \right),\;\lambda =-d,\;\lambda =-E,\;\lambda =-\left(d+\;\kappa \right), \end{array}$$

or


(9) $$\begin{array}{l} \lambda^{2}+\left[d+\theta -\left(\;\xi_{3}+d_{3}+d\right)\right]\lambda +\left(\;\xi_{3}+d_{3}+d\right)\left(d+\theta \right)\\ \;+\;\theta \xi_{3}=0. \end{array}$$

Here, all the eigenvalues except eigenvalues, with the exception of those in the expression of Equation 9 are negative, and for those in the expression (Equation 9) the Routh-Hurwitz stability criteria is applied and proved that the first column of the Routh-Hurwitz array has no sign change whenever${\;R}_{HB}<1$. Hence, the co-infection model disease-free equilibrium point (DFE) is locally asymptotically stable whenever${\;R}_{HB}<1$. The disease-free equilibrium of the model is locally asymptotically stable when the corresponding effective reproduction number is less than unity.

### Model endemic equilibrium point

The HBV and HIV co-infection model (4) endemic equilibrium point(s) is/are computed by making the right side of the system (4) equal to zero provided that *I*_*B*_ ≠ 0, or *I*_*H*_ ≠ 0, or *C* ≠ 0, or *T*_*B*_ ≠ 0, or *T* ≠ 0. Let the co-infection model (4) endemic equilibrium point be $E_{BH}^{*}\;=\left(S^{*},P^{*},\;I_{B}^{*},\;I_{H}^{*},\;C^{*},\;T_{B}^{*},\;T^{*}\;\right)$, and the forces of infection for HBV and HIV, respectively, are:

and ${\lambda_{B}}^{*}\left(t\right)=\frac{\beta_{1}}{N_{1}^{*}}\left(I_{B}^{*}\left(t\right)+\vartheta_{1}C^{*}\left(t\right)\right)$ and ${\lambda_{H}}^{*}\left(t\right)=\frac{\beta_{2}}{N_{2}^{*}}\left(I_{H}^{*}\left(t\right)+\vartheta_{2}C^{*}\left(t\right)\right).\;$ After solving and simplifying the result, we determined the following:


$$\begin{array}{l} S^{*}=\;\frac{\left(1-\tau \right)\text{Π}}{\left({\lambda_{H}}^{*}+{\lambda_{B}}^{*}+d\right)}+\frac{\nu }{\left({\lambda_{H}}^{*}+{\lambda_{B}}^{*}+d\right)}\;\left(\frac{\tau \text{Π}}{\left(\pi +d\right)}\right),\\ P^{*}=\frac{\tau \text{Π}}{\left(\pi +d\right)},I_{H}^{*}=\left(\frac{1}{\left(\eta_{1}{\lambda_{B}}^{*}+\xi_{2}+d_{2}+d\right)-\left(1-q\right)\nu }\right)\\ \;\left(\frac{\left(1-\tau \right)\text{Π}{\lambda_{H}}^{*}}{\left({\lambda_{H}}^{*}+{\lambda_{B}}^{*}+d\right)}+\frac{\pi {\lambda_{H}}^{*}}{\left({\lambda_{H}}^{*}+{\lambda_{B}}^{*}+d\right)}\left(\frac{{\lambda_{H}}^{*}\tau \text{Π}}{\left(\pi +d\right)}\right)\right),\\ I_{B}^{*}=(\frac{\left(\tau -1\right)\text{Π}{\lambda_{B}}^{*}}{\left({\lambda_{H}}^{*}+{\lambda_{B}}^{*}+d\right)}-\frac{\pi {\lambda_{Z}}^{*}}{\left({\lambda_{H}}^{*}+{\lambda_{B}}^{*}+d\right)}\\ \;\left(\frac{\tau \text{Π}}{\left(\pi +d\right)})\right)(\frac{\left(\kappa +d\right)}{\kappa \xi_{1}-\left(\kappa +d\right)(\eta_{2}{\lambda_{H}}^{*}+\xi_{1}+d_{1}+d)}),\\ T^{*}=\frac{\xi_{2}I_{H}^{*}+\xi_{3}C^{*}}{\left(\theta +d\right)},C^{*}=\frac{\eta_{1}{\lambda_{B}}^{*}I_{H}^{*}+\eta_{2}{\lambda_{H}}^{*}I_{B}^{*}+\theta T^{*}}{\left(\;\xi_{3}+m_{3}+d\;\right)},\\ T_{B}^{*}=\left(\frac{\xi_{1}}{\left(\theta +d\right)}\right)(\frac{\left(\tau -1\right)\text{Π}{\lambda_{B}}^{*}}{\left({\lambda_{H}}^{*}+{\lambda_{B}}^{*}+d\right)}-\frac{\pi {\lambda_{B}}^{*}}{\left({\lambda_{H}}^{*}+{\lambda_{B}}^{*}+d\right)}\\ \;(\frac{\tau \text{Π}}{(\pi +d)}))(\frac{(\kappa +d)}{\theta \xi_{1}-(\theta +d)\left(\eta_{2}{\lambda_{H}}^{*}+\xi_{1}+d_{1}+d\right)}). \end{array}$$

### Backward bifurcation for the co-infection model

Let *S* = *z*_1_, *P* = *z*_2_, *I*_*H*_ = *z*_3_, *I*_*B*_ = *z*_4_, *C* = *z*_5_, *T* = *z*_6_ and *T*_*B*_ = *z*_7_ and the total human population is given by *N* = *z*_1_ + *z*_2_ + *z*_3_ + *z*_4_ + *z*_5_ + *z*_6_ + *z*_7_ . Furthermore, using the vector representation $Z{\;=\left(z_{1},\;z_{2},\;z_{3},\;z_{4},z_{5},z_{6},z_{7}\right)}^{T},$ the HBV and HIV co-infection dynamical system (4) will be re-written as

$\frac{dZ}{dt}=H\left(Z\right)$ with $H={\left(h_{1},h_{2},h_{3},h_{4},h_{5},h_{6},h_{7},h_{8},h_{9}\right)}^{T}$ such that


(10) $$\begin{array}{lll} \frac{dz_{1}}{dt} & = & h_{1}=\left(1-\tau \right)\text{Π}+\pi z_{2}-\left(\lambda_{H}+\lambda_{B}+d\;\right)z_{1},\\ \frac{dz_{2}}{dt} & = & h_{2}=\tau \text{Π}-\left(\pi +d\;\right)z_{2},\\ \frac{dz_{3}}{dt} & = & h_{3}=\lambda_{H}z_{1}+\left(1-p\right)\text{ϕ}\nu z_{3}-\left(\eta_{1}\lambda_{B}+\xi_{2}+d_{2}+d\right)z_{3},\\ \frac{dz_{4}}{dt} & = & h_{4}=\lambda_{B}z_{1}+\kappa z_{7}-\left(\eta_{2}\lambda_{H}+\xi_{1}+d_{1}+d\right)z_{4},\\ \frac{dz_{5}}{dt} & = & h_{5}=\eta_{1}\lambda_{B}z_{3}+\eta_{2}\lambda_{H}z_{4}+\theta z_{6}-\left(\;\xi_{3}+d_{3}+d\right)z_{5},\\ \frac{dz_{6}}{dt} & = & h_{6}=\xi_{2}z_{3}+\xi_{3}z_{5}-\left(\theta +d\right)z_{6},\\ \frac{dz_{7}}{dt} & = & h_{7}=\xi_{1}z_{4}-\left(\kappa +d\;\right)z_{7}, \end{array}$$

where $\lambda_{B}\left(t\right)=\frac{\beta_{1}}{N\left(t\right)}\left(z_{4}\left(t\right)+\vartheta_{1}z_{5}\left(t\right)\right),$ and $\lambda_{H}\left(t\right)=\frac{\beta_{2}}{N\left(t\right)}\left(z_{3}\left(t\right)+\vartheta_{2}z_{5}\left(t\right)\right),$

for 1 ≤ ϑ_1_ < ∞ and 1 ≤ ϑ_2_ < ∞.

Then the Jacobian matrix of the new dynamical system given in Equation 10 at $E_{BH}^{0}$, represented by $J\left(E_{BH}^{0}\right)$

and determined by


$$\begin{array}{l} J\left(E_{BH}^{0}\right)=\left(\begin{array}{ccccccc} -d & \;\pi & \;-\frac{\beta_{2}}{N^{0}}z_{1}^{0} & -\frac{\beta_{1}}{N^{0}}z_{1}^{0} & -\frac{\beta_{1}}{N^{0}}{\vartheta_{1}z}_{1}^{0}-\frac{\beta_{2}}{N^{0}}\vartheta_{2}v_{1}^{0} & 0 & 0\\ 0 & -\left(\pi +d\right) & 0 & 0 & 0 & 0 & 0\\ 0 & 0 & {\;M}_{1} & 0 & \frac{\beta_{2}}{N^{0}}\vartheta_{2}v_{1}^{0} & 0 & 0\\ 0 & 0 & 0 & {\;M}_{2} & 0 & \kappa & 0\\ 0 & 0 & 0 & 0 & -\left(\;\xi_{3}+d_{3}+d\right) & \theta & 0\\ 0 & 0 & \xi_{2} & 0 & \xi_{3} & -\left(\theta +d\right) & 0\\ 0 & 0 & 0 & \xi_{1} & 0 & 0 & -\left(\kappa +d\right) \end{array}\right), \end{array}$$

where ${\;M}_{1}=\frac{\beta_{2}}{N^{0}}z_{1}^{0}+\left(1-p\right)\varphi \nu -\left(\xi_{2}+d_{2}+d\right)$,${\;M}_{2}=\frac{\beta_{1}}{N^{0}}z_{1}^{0}-\left(\xi_{1}+d_{1}+d\;\right).$

Let us assume that $R_{B}>R_{H}\;$without loss of the generality, and $R_{HB}=1$, i.e., $R_{B}=1$. Moreover, let $\beta_{1}\;=\beta^{*}\;$be a bifurcation parameter. Solving for *β*_1_ using $R_{B}=1$ as $R_{B}=\frac{\beta_{1}\pi +\beta_{1}d\left(1-\tau \right)}{\left(\pi +d\right)\left(\xi_{1}+d_{1}+d\right)}=1$ we determined as ${\beta^{*}=\beta }_{1}=\frac{\left(\pi +d\right)\left(\xi_{1}+d_{1}+d\right)}{\pi +d\left(1-\tau \;\right)}$.

Calculating the eigenvalues of the Jacobian matrix $J\left(E_{HB}^{0}\right)$ at the disease-free equilibrium point $E_{HB}^{0}$, for $\beta_{1}=\beta^{*}$,we determined the eigenvalues given by λ_1_ = −*d* < 0 or λ_2_ = −(π + *d*) < 0 or $\lambda_{3}={\;M}_{1}=\frac{\beta_{2}}{N^{0}}z_{1}^{0}+\left(1-p\right)\varphi \nu -\left(\xi_{2}+d_{2}+d\right)=\left(\xi_{2}+d_{2}+d\right)\left[R_{H}-1\right]<0$ if $R_{H}<1\;$or λ_4_ = 0 or λ_5_ = −(ξ_3_ + *d*_3_ + *d*) < 0 or λ_7_ = −(κ + *d*) < 0. From the results we observed that all the matrix $J\left(E_{HB}^{0}\right)$ eigenvalues are negative if $R_{HB}<1$. Then we applied the Center Manifold theory to investigate whether the HBV and HIV co-infection model (4) will or will not undergo the phenomenon of backward bifurcation at $R_{B}=1$. Now let us compute the eigenvectors of the Jacobian matrix $J_{\beta^{*}}$ at $R_{B}=1$. Then, based on the right eigenvectors $x={\left(x_{1},x_{2},x_{3},x_{4},x_{5},x_{6},x_{7}\right)}^{T}\;$and left eigenvectors *y* = (*y*_1_, *y*_2_, *y*_3_, *y*_4_, *y*_5_, *y*_6_, *y*_7_) of the Jacobian matrix $J_{\beta^{*}}$ at $\beta_{1}=\beta^{*}$ associated to the zero eigenvalue. After we computed and simplified the result, we have determined the bifurcation coefficients *a* and *b* such that


(11) $$\begin{array}{l} a=2y_{4}{x_{1}x}_{4}\frac{\partial^{2}h_{4}\left(0,0\right)}{\partial z_{1}\partial z_{4}}+2y_{4}{x_{3}x}_{4}\frac{\partial^{2}h_{4}\left(0,0\right)}{\partial z_{3}\partial z_{4}}\\ \;=2{\beta_{1}^{*}y}_{4}x_{4}\left[z_{1}+z_{2}\;\right],\\ \;=-2\beta_{2}^{*}{x_{4}y}_{4}^{2}\left[\frac{\beta_{1}z_{1}^{0}\left(\xi_{1}+d_{1}+d\right)+\left(\pi +d\right)\vartheta_{1}\beta_{1}z_{1}^{0}+d\left(\pi +d\right)\beta_{1}z_{2}^{0}}{d\left(\pi +d\right)\left(1-\tau \right)}\right]<0, \end{array}$$

and

$b=x_{4}y_{4}\frac{\partial^{2}h_{4}\left(0,0\right)}{\partial z_{4}\partial \beta_{1}}=x_{4}y_{4}\left(z_{1}^{0}+z_{2}^{0}\right)>0$, where *x*_4_ = *x*_4_ > 0, *y*_4_ = *y*_4_ > 0.

Therefore, according to the Castillo-Chavez and Song (50) criteria our proposed HBV and HIV co-infection model (4) does not exhibit bifurcation in the backward direction whenever $R_{B}=1.$ This result implies that in the co-infection model (4), the endemic equilibrium point (s) is/are does not exist in the region $R_{B}<1,$ meaning only the disease-free equilibrium point exists in the region $R_{B}<\;1$.

**Remark:** According to the results (values *a* < 0 and *b*>0) described in Equation 11 the HBV and HIV/AIDS co-infection model (4) disease-free equilibrium point $E_{BH}^{0}=\left(S^{0},P^{0},I_{B}^{0},I_{H}^{0},C^{0},T_{B}^{0},\;T^{0}\right)=\left(\frac{\pi \text{Π}+d\text{Π}\left(1-\tau \right)}{d\left(\pi +d\right)},\frac{\tau \text{Π}}{\pi +d},0,0,0,0,0\right)$ is globally asymptotically stable whenever $R_{HB}=\max\left\{\;R_{B},\;R_{H}\;\right\}<1$, where $R_{B}=\frac{\beta_{1}\pi +\beta_{1}d\left(1-\tau \right)}{\left(\pi +d\right)\left(\xi_{1}+d_{1}+d\right)}$ , is the HBV only infection effective reproduction number and${\;R}_{H}=\frac{\beta_{2}\left(\pi +d\left(1-\tau \right)\right)+\left(1-p\right)\nu \left(\pi +d\right)}{\left(\pi +d\right)\left(\xi_{2}+d_{2}+d\;\right)}.$

**Note:** From the investigation results of the bifurcation phenomenon above, it can be biologically (epidemiologically) suggested that the HBV and HIV co-infection disease may die out in the community whenever the HBV and HIV co-infection model disease-free equilibrium point is globally asymptotically stable whenever its effective reproduction number is less than one.

**Theorem 3:** The HBV and HIV co-infection model (4) disease-free equilibrium point $E_{HB}^{0}$ is globally asymptotically stable if the effective reproduction number $R_{HB}<1$ and is unstable if${\;R}_{HB}>\;1$.

### Sensitivity analysis

Since our study considers optimal control theory, we carried out the sensitivity analysis of the HBV and HIV co-infection model parameters incorporated in the effective reproduction numbers in this sub-section. Investigating the most influential model parameters that will increase or decrease the value of the threshold quantity (or the HBV and HIV co-infection model effective reproduction number${\;R}_{HB}$) is very crucial. Finding such influential model parameters that greatly impact the co-infection model is fundamental to reduce the spread of HBV and HIV co-infection spreading in the community. Using the model parameter values described in Table 1, we need to compute the sensitivity analysis of the model parameters using the HBV and HIV co-infection model effective reproduction number denoted by${\;R}_{HB}$ using the following well-known criteria:

**Definition:** Let *a* be an arbitrary model parameter incorporated in the HBV and HIV co-infection model (4) effective reproduction number${\;R}_{HB}$, then the forward sensitivity index formula is defined by $SI_{a}^{{\;R}_{HB}}=\frac{\partial {\;R}_{HB}}{\partial a}\times \frac{a}{{\;R}_{HB}}$ (7, 45).

Applying the HBV and HIV co-infection model parameters stated in Table 1, we have demined that ${\;R}_{H}=1.32>1$ and ${\;R}_{B}=1.14>1$ and also we have computed the values${\;R}_{HB}=\max\left\{{\;R}_{H},{\;R}_{B}\right\}={\;R}_{H}=1.32>1$. And also we computed the sensitivity indices as:


$$\begin{array}{l} \left(1\right).\;SI_{\pi }^{{\;R}_{H\;}}=\left(\frac{\partial {\;R}_{H\;}}{\partial \pi }\right)*\left(\frac{\pi }{{\;R}_{H\;}}\right)\Rightarrow SI_{\pi }^{{\;R}_{H\;}}\\ \;=\frac{\beta_{2}\pi \left(1-\pi \right)+\beta_{2}\pi d\tau }{\left(\pi +d\right)\left[\beta_{2}\left(\pi +d\left(1-\tau \right)\right)+\left(1-p\right)\nu \left(\pi +d\right)\right]}.\\ \left(2\right).\;SI_{\nu }^{{\;R}_{H\;}}=\left(\frac{\partial {\;R}_{H\;}}{\partial \nu }\right)*\left(\frac{\nu }{{\;R}_{H\;}}\right)\\ \;\Rightarrow SI_{\nu }^{{\;R}_{H\;}}=\frac{\left(1-p\right)\nu }{d+\left(1-p\right)\nu +d_{2}+\xi_{2}}>\;0.\\ \left(3\right).\;SI_{\beta_{2}}^{{\;R}_{H\;}}=\left(\frac{\partial {\;R}_{H\;}}{\partial \beta_{2}}\right)*\left(\frac{\beta_{2}}{{\;R}_{H\;}}\right)\Rightarrow \;SI_{\beta_{2}}^{{\;R}_{H\;}}\\ \;=\frac{\pi +d\left[1-\tau \right]}{\left(d+\pi \right)\left(d-\nu +p\nu +d_{2}+\xi_{2}\right)}\left(\frac{\beta_{2}}{{\;R}_{H\;}}\right)\\ \;=1>\;0.\\ \left(4\right).\;SI_{\tau }^{{\;R}_{H\;}}=\left(\frac{\partial {\;R}_{H\;}}{\partial \tau }\right)*\left(\frac{\tau }{{\;R}_{H\;}}\right)\\ \;\Rightarrow \;SI_{\tau }^{{\;R}_{H\;}}=\frac{-\beta_{2}d}{\left(\pi +d\right)\left(\xi_{2}+d_{2}+d\right)}<\;0.\\ \left(5\right).\;SI_{\pi }^{{\;R}_{B\;}}=\left(\frac{\partial {\;R}_{B\;}}{\partial \pi }\right)*\left(\frac{\pi }{{\;R}_{B\;}}\right)\\ \;\Rightarrow \;SI_{\pi }^{{\;R}_{B\;}}=\frac{\beta_{1}d\pi \tau }{\left(\pi +d\right)\left[\beta_{1}\pi +\beta_{1}d\left(1-\tau \right)\right]}>\;0.\\ \left(6\right).\;SI_{\beta_{1}}^{{\;R}_{B\;}}=\left(\frac{\partial {\;R}_{B\;}}{\partial \sigma }\right)*\left(\frac{\beta_{1}}{{\;R}_{B\;}}\right)\Rightarrow SI_{\beta_{1}}^{{\;R}_{B\;}}\\ \;=\frac{\pi +d\left[1-\tau \right]}{\left(d+\pi \right)\left(d+d_{1}+\xi_{1}\right)}\left(\frac{\beta_{1}}{{\;R}_{B\;}}\right)=1>\;0.\\ \left(7\right).\;SI_{\tau }^{{\;R}_{B\;}}=\left(\frac{\partial {\;R}_{B\;}}{\partial \tau }\right)*\left(\frac{\tau }{{\;R}_{B\;}}\right)\\ \;\Rightarrow SI_{\tau }^{{\;R}_{B\;}}=\frac{{-\beta }_{1}d\tau }{\beta_{1}\pi +\beta_{1}d\left(1-\tau \right)}<\;0.\\ \left(8\right).\;SI_{\xi_{1}}^{{\;R}_{B\;}}=\left(\frac{\partial {\;R}_{B\;}}{\partial \xi_{1}}\right)*\left(\frac{\xi_{1}}{{\;R}_{B\;}}\right)\\ \;\Rightarrow SI_{\xi_{1}}^{{\;R}_{B\;}}=\frac{-\left[\beta_{1}\pi +\beta_{1}d\left(1-\tau \right)\right]}{{\left(\xi_{1}+d_{1}+d\right)}^{2}}<\;0.\\ \left(9\right).\;SI_{\xi_{2}}^{{\;R}_{H\;}}=\left(\frac{\partial {\;R}_{H\;}}{\partial \xi_{2}}\right)*\left(\frac{\xi_{2}}{{\;R}_{H\;}}\right)=SI_{\xi_{2}}^{{\;R}_{H\;}}\\ \;=\frac{{-[\beta }_{2}(\pi +d\left(1-\tau )\right)+\left(1-p)\nu \left(\pi +d\right)\right]}{{(\xi_{2}+d_{2}+d)}^{2}}<\;0. \end{array}$$

Based on the model parameter values described in Table 1, we have determined the sensitivity index values represented in Tables 2, 3.

**Table 2:** Sensitivity indices whenever${\;R}_{HB}=\max\left\{{\;R}_{H},{\;R}_{B}\right\}={\;R}_{H}$.

**Parameters**	**Sensitivity indices**
*β*_2_	$SI_{\beta_{2}}^{R_{H\;}}=+1$
π	$SI_{\pi }^{R_{H\;}}=+0.21$
ν	$SI_{\nu }^{R_{H\;}}=+0.46$
τ	$SI_{\tau }^{R_{H\;}}=-0.82$
ξ_2_	$SI_{\xi_{2}}^{R_{H\;}}=-0.62$
*d*	$SI_{d}^{R_{H\;}}=+0.19$
*d*_2_	$SI_{d_{2}}^{R_{H\;}}=+0.36$

**Table 3:** Sensitivity indices whenever${\;R}_{HB}=\max\left\{{\;R}_{H},{\;R}_{B}\right\}={\;R}_{B}.$

**Parameters**	**Sensitivity indices**
*β*_2_	$SI_{\beta_{2}}^{R_{B}}=+1$
τ	$SI_{\tau }^{R_{B\;}}=-0.78$
π	$SI_{\pi }^{R_{B\;}}=+0.18$
ε_1_	$SI_{\varepsilon_{1}}^{R_{B}}=-0.65$
*d*	$SI_{d}^{R_{B}}=+0.21$
*d*_1_	$SI_{d_{1}}^{R_{B\;}}=+0.09$

Based on the sensitivity indices described in Tables 2, 3, we have the following diagrams that show the graphical representations of the values represented therein.

The diagrams shown in Figures 2A, B are carried out by considering $R_{HB}>1$ mean that, when the HBV and HIV co-infection disease spreads throughout the community. The results reveal that the diseases transmission rates and portion of protection have the most significant impact on the co-infection model effective reproduction number.

### Optimal control problem and its analysis

In this section, based on the HBV and HIV co-infection spreading model (4) parameters sensitivity indices described in Tables 2, 3 above and the associated sensitivity indices diagram shown in Figures 2A, B above we re-constructed the optimal control problem (12) by considering the bounded, Lebesgue integrable control functions, denoted by *c* = (*c*_1_, *c*_2_, *c*_3_, *c*_4_, *c*_5_) such that

- The control function *c*_1_(*t*) represent efforts to protect individuals against HIV infection by using education and condom use,
- The control function *c*_2_(*t*) represent efforts to protect individuals against HBV infection by using education and condom use,
- The control function *c*_3_(*t*) is the control related to treatment of HIV infected individuals to increase their recovery rate and recovery period.
- The control function *c*_4_(*t*) is the control related to treatment of HBV infected individuals to increase their recovery rate and recovery period,
- The control function *c*_5_(*t*) is the control related to treatment of the HIV and HBV co-infected individuals to increase their recovery rate and recovery period.

Thus, for this particular study the implementation of the right protection and treatment strategies on the HBV and HIV co-infected individuals, HBV and HIV single infected individuals in a community is used to improve the recovery period and increase the number of recovered individuals such that 0 ≤ *c*_1_, *c*_2_, *c*_3_, *c*_4_, *c*_5_ ≤ 1. After incorporating all these five control strategies described above in to the dynamical system (4) the corresponding system of differential equation for the HBV and HIV co-infection transmission model (4) can be re-written as follows:


(12) $$\begin{array}{lll} \frac{dS}{dt} & = & \;\left(1-q\right)\text{Π}+\pi P-\left(\left(1-c_{1}\left(t\right)\right)\lambda_{H}+\left(1-c_{2}\left(t\right)\right)\lambda_{B}+d\right)S,\\ \frac{dP}{dt} & = & q\text{Π}-\left(\pi +d\right)P,\\ \frac{dI_{H}}{dt} & = & \left(1-c_{1}\left(t\right)\right)\lambda_{H}S+\left(1-\pi \right)\nu I_{H}-\left(\eta_{2}\lambda_{H}+c_{3}\left(t\right)\xi_{2}+d_{2}+d\;\right)I_{H},\\ \frac{dI_{B}}{dt} & = & \left(1-c_{2}\left(t\right)\right)\lambda_{B}S+\kappa T-\left(\eta_{1}\lambda_{H}+{c_{4}\left(t\right)\;\xi }_{1}+d_{1}+d\right)I_{B},\\ \frac{dC}{dt} & = & \eta_{1}\lambda_{B}I_{H}+\eta_{1}\lambda_{H}I_{B}+\rho T-\left(\;c_{5}\left(t\right)\xi_{3}+d_{3}\;+d\right)C,\\ \frac{dT}{dt} & = & {c_{3}\left(t\right)\xi }_{2}I_{H}+c_{5}\left(t\right)\xi_{3}C-\left(\theta +d\right)T,\\ \frac{dT_{B}}{dt} & = & c_{4}\left(t\right)\xi_{1}I_{B}-\left(\kappa +d\right)T_{B}, \end{array}$$

subject to the initial conditions *S*(0) = *S*^0^ ≥ 0, *P*(0) = *P*^0^ ≥ 0, $I_{H}\left(0\right)={I_{H}}^{0}\geq 0$, $I_{B}\left(0\right)={I_{B}}^{0}\geq 0$, *C*(0) = *C*^0^ ≥ 0, *T*(0) = *T*^0^ ≥ 0, and $T_{B}\left(0\right)={T_{B}}^{0}\geq \;0$.

The objective functional is represented by


(13) $$\begin{array}{l} J\left(c_{1},c_{2},c_{3},c_{4},c_{5}\right)\;=\int_{0}^{T_{f}}\left(\varphi_{1}I_{H}+\varphi_{2}I_{B}+\varphi_{3}C+\frac{1}{2}\overset{5}{\underset{i=1}{\sum }}{\text{ϕ}}_{i}c_{i}^{2}\right)dt, \end{array}$$

where *T*_*f*_ is the final time φ_1_, φ_2_ and φ_3_ are weight constants of the HIV infected individuals, the HBV infected and the HIV and HBV co-infected individuals respectively while ϕ_*i*_ for *i* = 1, ..., 5, are weight constants for each individual time-dependent control strategy. We choose a nonlinear cost on the control strategies based on the assumption that the costs take nonlinear form as applied in references (34, 36, 37).

The main objective in this section is to find the optimal control strategies $c_{1}^{*},\;c_{2}^{*},c_{3}^{*},\;c_{4}^{*},c_{5}^{*}$ subjected to Equation 12 such that


(14) $$\begin{array}{l} \;J\left(c_{1}^{*},\;c_{2}^{*},c_{3}^{*},\;c_{4}^{*},c_{5}^{*}\;\right)\\ =\min\left\{J\left(c_{1},c_{2},c_{3},c_{4},c_{5}\right):\;c_{1},c_{2},c_{3},c_{4},c_{5}\in c\right\}, \end{array}$$

where $c=\left\{c_{1}\left(t\right),\;c_{2}\left(t\right),c_{3}\left(t\right),\;c_{4}\left(t\right),\;c_{5}\left(t\right)\in {\mathbb{R}}^{5}\right\}$ such that *c*_1_(*t*), *c*_2_(*t*), *c*_3_(*t*), *c*_4_(*t*), *c*_5_(*t*) are Lebesgue measurable functions and 0 ≤ *c*_1_(*t*), *c*_2_(*t*), *c*_3_(*t*), *c*_4_(*t*), *c*_5_(*t*) ≤ 1 for 0 ≤ *t* ≤ *T*_*f*_ is the control set.      (15)

**Theorem 4:** Given the cost functional illustrated by *J*(*c*_1_, *c*_2_, *c*_3_, *c*_4_, *c*_5_) subject to the system of Equation 13, then there exist an optimal control function $c^{*}=\left(c_{1}^{*},\;c_{2}^{*},c_{3}^{*},\;c_{4}^{*},c_{5}^{*}\right)$ and corresponding optimal solutions to the initial value problem (Equations 12–**15**) with the model optimal solution $\left(S^{*},P^{*},I_{H}^{*},I_{B}^{*},\;C^{*},\;T_{B}^{*},\;T^{*}\right)$, that minimizes *J*(*c*_1_, *c*_2_, *c*_3_, *c*_4_, *c*_5_) over *c*.

**Proof:** To verify the following four basic conditions required for the set of admissible controls *c* we can use the Fleming and Rishel's theorem stated in (40).

*I*: The set of the model state variables to the system (Equations 12–**15**) that correspond to the control functions in *c* is non-empty.

*II*: The control set c is closed and convex.

*III*: Each right-hand side of the state system is continuous, is bounded above by a sum of the bounded control and the state, and can be written as a linear function of *c* = (*c*_1_, *c*_2_, *c*_3_, *c*_4_, *c*_5_) with coefficients depending on time and the state.

*IV*: The integrand of the objective functional given in Equation 13 is convex.

The first required condition (*I*) can be verified by using Picard-Lindelöf's theorem. If the solutions to the co-infection dynamical system equations solutions are bounded, continuous and satisfies Lipschitz conditions in the model state variables, then there is a unique model solution corresponding to each admissible control function (strategy) in the control set *c*. We have proved that the total number of human population at time *t* is bounded as $0\leq N\left(t\right)\leq \frac{\text{Π}}{d}$ also each of the model state variables is bounded. Hence the model state variables are continuous and bounded. Similarly we can prove the boundedness of the partial derivatives with respect to the state variables in the model, which establishes that the model is Lipschitz with respect to the co-infection model state variables. This completes the verification that condition *I* holds.

By applying the definition stated in references (41–43), the control set *c* is convex and closed, this proved the required condition *II*. Condition *III* is verified by observing the linear dependence of the model equations on the control variables *c*_1_, *c*_2_, *c*_3_, *c*_4_, *c*_5_.

Eventually, to justify the required condition *IV* use definition stated in (40, 43) that says any constant, linear and quadratic functions are convex. Hence, since the integrand of the objective functional given by $T\left(x,\;c,\;t\right)=\varphi_{1}I_{H}+\varphi_{2}I_{B}+\varphi_{3}C+\frac{1}{2}{\text{ϕ}}_{1}c_{1}^{2}+\frac{1}{2}{\text{ϕ}}_{2}c_{2}^{2}+\frac{1}{2}{\text{ϕ}}_{3}c_{3}^{2}+\frac{1}{2}{\text{ϕ}}_{4}c_{4}^{2}+\frac{1}{2}{\text{ϕ}}_{5}c_{5}^{2}$is a quadratic function that is convex on *c*. To show the bound on *T*(*x, c, t*) use definition of the control function *c* as: then we have $\frac{1}{2}{\text{ϕ}}_{5}c_{5}^{2}\leq \frac{1}{2}{\text{ϕ}}_{5}$ since 0 ≤ *c*_5_ ≤ 1 and hence $\frac{1}{2}\omega \varphi_{5}c_{5}^{2}-\frac{1}{2}{\text{ϕ}}_{5}\leq \;0.$


$$\begin{array}{l} \Rightarrow T\left(x,\;c,\;t\right)=\varphi_{1}I_{H}+\varphi_{2}I_{B}+\varphi_{3}C+\frac{1}{2}\phi_{1}c_{1}^{2}+\frac{1}{2}\phi_{2}c_{2}^{2}\\ \;+\frac{1}{2}\phi_{3}c_{3}^{2}+\frac{1}{2}\phi_{4}c_{4}^{2}+\frac{1}{2}\phi_{5}c_{5}^{2}\geq \frac{1}{2}\phi_{1}c_{1}^{2}+\frac{1}{2}\phi_{2}c_{2}^{2}\\ \;+\frac{1}{2}\phi_{3}c_{3}^{2}+\frac{1}{2}\phi_{4}c_{4}^{2}+\frac{1}{2}\phi_{5}c_{5}^{2}-\;\frac{1}{2}\phi_{5}.\\ \Rightarrow T\left(x,\;c,\;t\right)\geq \min\left\{\frac{1}{2}\phi_{1},\frac{1}{2}\phi_{2},\frac{1}{2}\phi_{3},\frac{1}{2}\phi_{4},\frac{1}{2}\phi_{5}\right\}\\ \;\left(c_{1}^{2}+c_{2}^{2}+c_{3}^{2}+c_{4}^{2}+c_{5}^{2}\right)-\;\frac{1}{2}\phi_{5}.\\ \Rightarrow T\left(x,\;c,\;t\right)\geq \min\left\{\frac{1}{2}\phi_{1},\frac{1}{2}\phi_{2},\frac{1}{2}\phi_{3},\frac{1}{2}\phi_{4},\frac{1}{2}\phi_{5}\right\}\\ \;\left(|c_{1},c_{2},c_{3},c_{4},c_{5}\right){|}^{2})-\;\frac{1}{2}\phi_{5}.\\ \Rightarrow T\left(x,\;c,\;t\right)\geq M_{1}|c{|}^{\beta }-M_{2},\text{ where }M_{1}\\ \;=\min\left\{\frac{1}{2}\phi_{1},\frac{1}{2}\phi_{2},\frac{1}{2}\phi_{3},\frac{1}{2}\phi_{4},\frac{1}{2}\phi_{5}\right\},M_{2}=\frac{1}{2}\phi_{5}, \end{array}$$

*c* = (*c*_1_, *c*_2_, *c*_3_, *c*_4_, *c*_5_), and *β* = 2. This completes the proof of Theorem 8 stated above.

The necessary conditions that an optimal solution must satisfy come from Pontryagin's minimum principle (PMP). This principle converts (Equation 12–14) in to a problem of minimizing a Hamiltonian, H with respect to *c*_1_, *c*_2_, *c*_3_, *c*_4_, *c*_5_ together with the state equation and the adjoint condition.

The Hamiltonian function is illustrated by


(16) $$\begin{array}{l} H=\varphi_{1}I_{H}+\varphi_{2}I_{B}+\varphi_{3}C+\frac{1}{2}\phi_{1}c_{1}^{2}+\frac{1}{2}\phi_{2}c_{2}^{2}+\frac{1}{2}\phi_{3}c_{3}^{2}+\frac{1}{2}\phi_{4}c_{4}^{2}\\ \;+\frac{1}{2}\phi_{5}c_{5}^{2}\\ \;+\delta_{1}(\left(1-\tau \right)\text{Π}+\pi P(\left(1-c_{1}\left(t\right)\right)\lambda_{H}\\ \;+\left(1-c_{2}\left(t\right)\right)\lambda_{B}+d\;)S)\\ \;+\delta_{2}\left(\tau \text{Π}-\left(\pi +d\;\right)P\right)\\ \;+\delta_{3}(\left(1-c_{1}\left(t\right)\right)\lambda_{H}S+\left(1-\pi \right)\nu I_{H}\\ \;-\left(\eta_{2}\lambda_{H}+c_{3}\left(t\right)\xi_{2}+d_{2}+d\right)I_{H})\\ \;+\delta_{4}\left(\left(1-c_{2}\left(t\right)\right)\lambda_{B}S+\kappa T-\left(\eta_{2}\lambda_{H}+{c_{4}\left(t\right)\xi }_{1}+d_{1}+d\;\right)I_{B}\right)\\ \;+\delta_{5}\left(\eta_{1}\lambda_{B}I_{H}+\eta_{2}\lambda_{H}I_{B}+\rho T\left(\;c_{5}\left(t\right)\phi_{3}+d_{3}+d\;\right)C\right)\\ \;+\delta_{6}\left({c_{3}\left(t\right)\xi }_{2}I_{H}+c_{5}\left(t\right)\xi_{3}C-\left(\theta +d\;\right)T\right)\\ \;+\delta_{7}\left(c_{4}\left(t\right)\phi_{1}I_{B}-\left(\kappa +d\right)T\right), \end{array}$$

where δ_*i*_, *i* = 1, …, 7 are the adjoint variables.

**Theorem 5:** For an optimal control set *c*_1_, *c*_2_, *c*_3_, *c*_4_, *c*_5_ that minimizes *J* over *c*, there is an adjoint variables, δ_1_, ..., δ_7_ such that:


(17) $$\begin{array}{l} \frac{d\delta_{1}}{dt}=\left(\delta_{1}-\delta_{3}\right)\left(1-c_{1}\left(t\right)\right)\left(\frac{\beta_{2}\left(I_{H}\left(t\right)+\vartheta_{2}C\left(t\right)\right)N-\beta_{1}\left(I_{H}\left(t\right)+\vartheta_{2}C\left(t\right)\right)S}{N^{2}}\right)\\ \;+{\;(\delta }_{1}-\delta_{4})\left(1-c_{2}\left(t\right)\right)(\frac{\beta_{1}\left(I_{B}\left(t\right)+\vartheta_{1}C\left(t\right)\right)N-\beta_{1}(I_{B}\left(t\right)+\vartheta_{1}C\left(t\right))S}{N^{2}})\\ \;+\delta_{1}d+\left(\delta_{5}-\delta_{4}\right)\eta_{1}\beta_{1}\left(\frac{I_{H}\left(t\right)+\vartheta_{2}C\left(t\right)}{N^{2}}\;\right)I_{B}\\ \;+\left(\delta_{5}-\delta_{3}\right)\eta_{2}\;\beta_{1}\left(\frac{I_{B}\left(t\right)+\vartheta_{1}C\left(t\right)}{N^{2}}\right)I_{H}\;+\;\delta_{1}d,\\ \frac{d\delta_{2}}{dt}=\left(\delta_{2}-\delta_{1}\right)\pi +\;\delta_{2}d,\\ \frac{d\delta_{3}}{dt}=\left(\delta_{1}-\delta_{3}\right)\left(1-c_{1}\left(t\right)\right)\left(\frac{\beta_{2}SN-\alpha_{1}\left(I_{H}\left(t\right)+\vartheta_{2}C\left(t\right)\right)S}{N^{2}}\right)\\ \;+\left(\delta_{4}-\delta_{1}\right)\beta_{1}\left(1-c_{2}\left(t\right)\right)\left(\frac{\left(I_{B}\left(t\right)+\vartheta_{1}C\left(t\right)\;\right)S}{N^{2}}\right)+\\ \;+\left(\delta_{4}-\delta_{5}\right){\beta_{2}\eta }_{1}\left(\frac{N-\left(I_{H}\left(t\right)+\vartheta_{2}C\left(t\right)\right)I_{B}}{N^{2}}\right)\\ \;+\left(2\delta_{3}-\delta_{5}\right)\beta_{1}\eta_{2}\left(\frac{\left(I_{B}\left(t\right)+\vartheta_{1}C\left(t\right)\right)N-\left(I_{B}\left(t\right)+\vartheta_{1}C\left(t\right)\right)I_{H}}{N^{2}}\right)\\ \;+\left(\delta_{3}-\delta_{6}\right)c_{3}\left(t\right)\xi_{2}+\delta_{3}\left(d_{2}+d-\left(1-\pi \right)\nu \right)-\;\varphi_{1},\\ \frac{d\delta_{4}}{dt}=\left(\delta_{3}-\delta_{1}\right)\left(1-u_{1}\left(t\right)\right)\beta_{2}\frac{\left(I_{H}\left(t\right)+\vartheta_{2}C\left(t\right)\right)S}{N^{2}}\\ \;+\left(\delta_{1}-\;\delta_{4}\right)\left(1-u_{2}\left(t\right)\right)\beta_{1}\left(\frac{NS-\left(I_{B}\left(t\right)+\vartheta_{1}C\left(t\right)\right)S}{N^{2}}\right)\\ \;+(\delta_{4}{-\delta_{5})\eta }_{1}\beta_{2}\left(\frac{\left(I_{H}\left(t\right)+\vartheta_{2}C\left(t\right)\right)N-\left(I_{B}\left(t\right)+\vartheta_{2}C\left(t\right)\right)I_{B}}{N^{2}}\right)\\ \;+\left(\delta_{3}-\delta_{5}\right)\eta_{2}\beta_{1}\left(\frac{NI_{H}-\left(I_{B}\left(t\right)+\vartheta_{1}C\left(t\right)\right)I_{H}}{N^{2}}\right)\\ \;+\left(\delta_{4}-\delta_{7}\right){c_{4}\left(t\right)\xi }_{1}+\delta_{4}\left(d_{1}+d\right)-\;\varphi_{2},\\ \frac{d\delta_{5}}{dt}={(\delta }_{1}-\delta_{3})\left(1-c_{1}\left(t\right)\right)\beta_{2}(\frac{\vartheta_{2}NS-(I_{H}(t)+\vartheta_{2}C(t))S}{N^{2}})\\ \;+\left(\delta_{1}-\delta_{4}\right)\left(1-c_{2}\left(t\right)\right)\beta_{1}\left(\frac{\vartheta_{1}NS-\left(I_{B}\left(t\right)+\vartheta_{1}C\left(t\right)\right)S}{N^{2}}\right)\\ \;+{(\delta }_{4}-\delta_{5})\eta_{1}\beta_{2}(\frac{\vartheta_{2}NP-(I_{H}(t)+\vartheta_{2}C(t))I_{B}}{N^{2}})\\ \;+\left(\delta_{3}-\delta_{5}\right)\eta_{2}\beta_{1}\left(\frac{\vartheta_{1}NI_{H}-\left(I_{B}\left(t\right)+\vartheta_{1}C\left(t\right)\right)I_{H}}{N^{2}}\right)\\ \;++\left(\delta_{5}-\delta_{6}\right){\;c}_{5}\left(t\right)\xi_{3}+\delta_{5}\left(d_{3}+d\right)-\;\varphi_{3},\\ \frac{d\delta_{6}}{dt}=\left(\delta_{3}-\delta_{1}\right)\left(1-c_{1}\left(t\right)\right)\beta_{2}\frac{\left(I_{H}\left(t\right)+\vartheta_{2}C\left(t\right)\right)S}{N^{2}}\\ \;++\left(\delta_{4}-\delta_{1}\right)\left(1-c_{2}\left(t\right)\right)\beta_{1}\frac{\left(I_{B}\left(t\right)+\vartheta_{1}C\left(t\right)\right)S}{N^{2}}\\ \;+(\delta_{5}-\delta_{4}{)\eta }_{1}\beta_{2}\frac{\left(I_{H}\left(t\right)+\vartheta_{2}C\left(t\right)\right)I_{B}}{N^{2}}\\ \;+\left(\delta_{5}-\delta_{3}\right)\eta_{2}\beta_{1}\frac{\left(I_{B}\left(t\right)+\vartheta_{1}C\left(t\right)\right)I_{H}}{N^{2}}\\ \;+\left(\delta_{6}-\;\delta_{5}\right)\rho +\delta_{6}d,\\ \frac{d\delta_{7}}{dt}=\left[\left(\delta_{3}-\delta_{1}\right)\right]\beta_{2}\frac{\left(I_{H}\left(t\right)+\vartheta_{2}C\left(t\right)\right)S}{N^{2}}\\ \;+\left(\delta_{4}-\delta_{1}\right)\left(1-c_{2}\left(t\right)\right)\beta_{1}\frac{\left(I_{B}\left(t\right)+\vartheta_{1}C\left(t\right)\right)S}{N^{2}}\\ \;+(\delta_{5}-\delta_{4}{)\eta }_{1}\beta_{2}\frac{\left(I_{H}\left(t\right)+\vartheta_{2}C\left(t\right)\right)I_{B}}{N^{2}}\\ \;+\left(\delta_{5}-\delta_{3}\right)\eta_{2}\beta_{1}\frac{\left(I_{B}\left(t\right)+\vartheta_{1}C\left(t\right)\right)I_{H}}{N^{2}}+\left(\delta_{7}-\;\delta_{4}\right)\sigma +\delta_{7}d, \end{array}$$

with the final conditions δ_*i*_(*T*_*f*_) = 0, for *i* = 1, …, 7.

**Proof:** Let us use the necessary and sufficient conditions stated as


(18) $$\begin{array}{r} \frac{d\delta_{1}}{dt}=-\frac{\partial H}{\partial S},\frac{d\delta_{4}}{dt}=-\frac{\partial H}{\partial I_{B}},\\ \frac{d\delta_{2}}{dt}=-\frac{\partial H}{\partial P},\frac{d\delta }{dt}=-\frac{\partial H}{\partial C},\\ \frac{d\delta_{3}}{dt}=-\frac{\partial H}{\partial I_{H}},\frac{d\delta_{6}}{dt}=-\frac{\partial H}{\partial T},\text{and}\frac{d\delta_{7}}{dt}=-\frac{\partial H}{\partial T}. \end{array}$$

Then solving the results of Equation 18 the full expression of the adjoint functions $\frac{d\delta_{i}}{dt}\;$for *i* = 1, 2, 3, 4, 5, 6, 7 of the optimal control system (Equation 12) based on Equations 15, 16 are given by the system (Equation 17). This completes the proof of the theorem.

Optimality Conditions: The first conditions that we will consider from the Pontryagin's Maximum/Minimum principle applied in (44) are the minimization of the Hamiltonian H with respect to the control functions *c*_1_, *c*_2_, *c*_3_, *c*_4_, *c*_5_. Since the cost function is convex, if the optimal control occurs in the interior region we must have the following basic necessary and sufficient optimality conditions for the optimal control problem (Equation 12) as:


(19) $$\begin{array}{l} \frac{\partial H}{\partial c_{1}^{*}}=0,\;\frac{\partial H}{\partial c_{2}^{*}}=0,\;\frac{\partial H}{\partial c_{3}^{*}}=0,\;\frac{\partial H}{\partial c_{4}^{*}}=0,\;and\;\frac{\partial H}{\partial c_{5}^{*}}=0. \end{array}$$

After solving and simplifying the results computed from Equation 19 we have determined the final optimal control strategies results given by:


(20) $$\begin{array}{r} c_{1}^{*}=\max\;\left\{0,\;\min\;\left\{1,\frac{(\delta_{3}-\;\delta_{1}{)\lambda }_{H}S}{\phi_{1}}\right\}\right\},\\ c_{2}^{*}=\max\;\left\{0,\;\min\;\left\{1,\frac{(\delta_{4}-\delta_{1}{)\lambda }_{B}S}{\phi_{2}}\right\}\right\},\\ c_{3}^{*}=\max\;\left\{0,\;\min\;\left\{1,\frac{(\delta_{3}-\;\delta_{6}{)\xi }_{2}I_{H}}{\phi_{3}}\right\}\right\},\\ c_{4}^{*}=\max\;\left\{0,\;\min\left\{1,\frac{(\delta_{4}-\;\delta_{7}{)\xi }_{1}I_{B}}{\phi_{4}}\right\}\right\},\\ c_{5}^{*}=\max\;\left\{0,\;\min\;\left\{1,\frac{(\delta_{5}-\;\delta_{6}{)\xi }_{3}I_{B}C}{\phi_{5}}\right\}\right\}. \end{array}$$

**Theorem 6:** For any *t* ∈ [0, *T*_*f*_], the bounded solutions (Equation 20) to the optimality system are unique, and we can refer to (42), for the proof of the theorem.

## Results and discussions

In this subsection, we simulate the optimal control system (Equation 12) using the Runge–Kutta fourth order forward-backward sweep method with MATLAB, whose accuracy, convergence, and stability have been proved by Lenhart and Worksman (47) and taking into consideration seven possible combination control strategies presented below. The RK4 technique evaluates the derivative function at several intermediate points within the step interval in order to determine the values of the dependent variables at each step. To obtain an estimation of the derivative at the currrent stage, it then weights averages these intermediate evaluations. The dependent variable values are updated using this estimate, and the process is repeated iteratively until the intended endpoint is reached. By considering multiple intermediate evaluations, the RK4 method provides a more accurate approximation compared to other simple numerical methods, making it a popular choice for numerical ODE integration. Numerical simulations of the optimal control problem (Equation 12), a critical aspect of this manuscript, offering a computational approach to solve complex systems where analytical solutions are often impractical. Optimal control requires determining the best control inputs over time to achieve a desired objective while adhering to constraints. Moreover, numerical simulations of the optimal control problem enhance the manuscript by providing insights into the system dynamics, performance optimization, and the robustness of control strategies across various applications. The inclusion of an optimal control framework in the research is of paramount significance, as it introduces five controls designed to manage the dynamics of HBV and HIV co-infection. These controls include strategies to prevent HBV and HIV infections, improve recovery from infection, and provide treatment for co-infected individuals. The section focuses on the critical importance of these control strategies, both collectively and individually, underscoring their role in shaping effective approaches to address the complexities of HBV and HIV co-infection dynamics. To verify the effect of the proposed control strategies and verify the analytical results of the optimal control problem (Equation 12), we carried out a numerical simulation by considering the following equal weight factors, with the assumptions of different initial population for the state variables along with the parameter values described in Table 2, such that φ_1_ = φ_2_ = φ_3_ = 10, ϕ_1_ = ϕ_2_ = ϕ_3_ = 15.

We proposed the following control strategies with five different scenarios by:

(i). **Scenario A** (using one control measure only):

Strategy 1: Applying *c*_1_≠0, *c*_2_ = *c*_3_ = *c*_4_ = *c*_5_ = 0,

Strategy 2: Applying *c*_2_≠0, *c*_1_ = *c*_3_ = *c*_4_ = *c*_5_ = 0,

Strategy 3: Applying *c*_3_≠0, *c*_1_ = *c*_2_ = *c*_4_ = *c*_5_ = 0,

Strategy 4: Applying *c*_4_≠0, *c*_1_ = *c*_2_ = *c*_3_ = *c*_5_ = 0,

Strategy 5: Applying *c*_5_≠0, *c*_1_ = *c*_2_ = *c*_3_ = *c*_4_ = 0.

(ii). **Scenario B** (using two control measures):

Strategy 6: Applying *c*_1_≠0, *c*_2_≠0, *c*_3_ = *c*_4_ = *c*_5_ = 0,

Strategy 7: Applying *c*_1_≠0, *c*_3_≠0, *c*_2_ = *c*_4_ = *c*_5_ = 0,

Strategy 8: Applying *c*_1_≠0, *c*_4_≠0, *c*_2_ = *c*_3_ = *c*_5_ = 0,

Strategy 9: Applying *c*_1_≠0, *c*_5_≠0, *c*_2_ = *c*_3_ = *c*_4_ = 0,

Strategy 10: Applying *c*_2_≠0, *c*_3_≠0, *c*_1_ = *c*_4_ = *c*_5_ = 0,

Strategy 11: Applying *c*_2_≠0, *c*_4_≠0, *c*_1_ = *c*_3_ = *c*_5_ = 0,

Strategy 12: Applying *c*_2_≠0, *c*_5_≠0, *c*_1_ = *c*_3_ = *c*_4_ = 0,

Strategy 13: Applying *c*_3_≠0, *c*_4_≠0, *c*_1_ = *c*_2_ = *c*_5_ = 0,

Strategy 14: Applying *c*_3_≠0, *c*_5_≠0, *c*_1_ = *c*_2_ = *c*_4_ = 0,

Strategy 15: Applying *c*_4_≠0, *c*_5_≠0, *c*_1_ = *c*_2_ = *c*_3_ = 0.

(iii). **Scenario C** (using three control measures):

Strategy 16: Applying *c*_1_≠0, *c*_2_≠0, *c*_3_≠0, *c*_4_ = *c*_5_ = 0,

Strategy 17: Applying *c*_1_≠0, *c*_2_≠0, *c*_4_≠0, *c*_3_ = *c*_5_ = 0,

Strategy 18: Applying *c*_1_≠0, *c*_2_≠0, *c*_5_≠0, *c*_3_ = *c*_4_ = 0,

Strategy 19: Applying *c*_1_≠0, *c*_3_≠0, *c*_4_≠0, *c*_2_ = *c*_5_ = 0,

Strategy 20: Applying *c*_1_≠0, *c*_3_≠0, *c*_5_≠0, *c*_2_ = *c*_4_ = 0,

Strategy 21: Applying *c*_1_≠0, *c*_4_≠0, *c*_5_≠0, *c*_1_ = *c*_3_ = 0,

Strategy 22: Applying *c*_2_≠0, *c*_3_≠0, *c*_4_≠0, *c*_1_ = *c*_5_ = 0,

Strategy 23: Applying *c*_2_≠0, *c*_3_≠0, *c*_5_≠0, *c*_2_ = *c*_4_ = 0,

Strategy 24: Applying *c*_2_≠0, *c*_4_≠0, *c*_5_≠0, *c*_1_ = *c*_3_ = 0,

Strategy 25: Applying *c*_3_≠0, *c*_4_≠0, *c*_5_≠0, *c*_1_ = *c*_2_ = 0.

(iv). **Scenario D** (using four control measures):

Strategy 26: Applying *c*_1_≠0, *c*_2_≠0, *c*_3_≠0, *c*_4_≠0, *c*_5_ = 0,

Strategy 27: Applying *c*_1_≠0, *c*_2_≠0, *c*_3_≠0, *c*_5_≠0, *c*_4_ = 0,

Strategy 28: Applying *c*_1_≠0, *c*_3_≠0, *c*_4_≠0, *c*_5_≠0, *c*_2_ = 0,

Strategy 29: Applying *c*_1_≠0, *c*_2_≠0, *c*_4_≠0, *c*_5_≠0, *c*_3_ = 0,

Strategy 30: Applying, *c*_2_≠0, *c*_3_≠0, *c*_4_≠0, *c*_5_ = 0, *c*_1_ = 0.

(v). **Scenario E** (using five control measures):

Strategy 31: Applying *c*_1_≠0, *c*_2_≠0, *c*_3_≠0, *c*_4_≠0, *c*_5_≠ 0.

### Simulations for Scenario A

**Figure 3:**
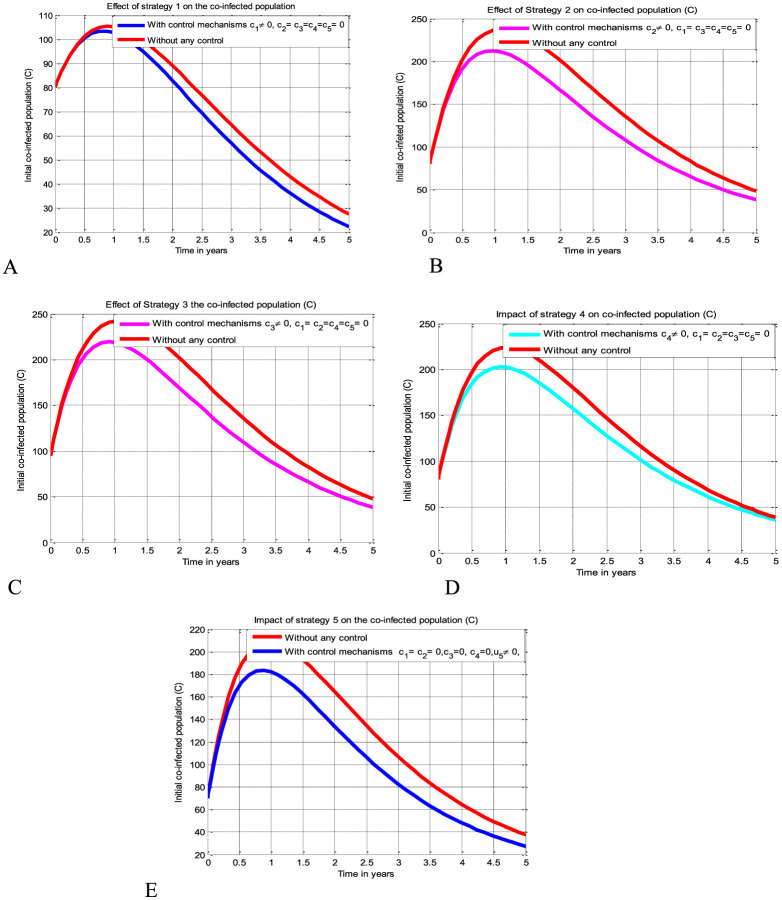
Potential impact of single strategies on the HBV and HIV co-infection disease. **(A)** Impact of HIV protection on the co-infection. **(B)** Impact of HBV protection on the co-infection. **(C)** Impact of HIV treatment on the co-infection. **(D)** Impact of HBV treatment on the co-infection. **(E)** Impact of simultaneous HBV and HIV treatments on the co-infection.

The simulation curve shown in Figure 3A suggests the impact of the control measure *c*_1_ (i.e., the potential impact of implementing the HIV protection control mechanism), emphasizing a significant reduction of the HBV and HIV co-infected population as compared to a scenario where there is no control mechanism implemented. The simulation curve illustrated in Figure 3B shows the impact of the control measure *c*_2_ (i.e., the possible impact of implementing the HBV protection control mechanism) emphasizing a significant reduction of the HBV and HIV co-infected population as compared to a scenario where there is no control mechanism implemented. The simulation curve illustrated in Figure 3C shows the impact of the control measure *c*_3_ (i.e., the possible impact of implementing the HIV treatment control mechanism), emphasizing a significant reduction in the HBV and HIV co-infected population as compared to a scenario where there is no control mechanism implemented. The simulation curve illustrated in Figure 3D shows the impact of the control strategy *c*_4_ (i.e., impact of implementing the HBV treatment control mechanism), emphasizing a significant reduction in the HBV and HIV co-infected population as compared to a scenario where there is no control mechanism implemented. The simulation curve illustrated in Figure 3E shows the impact of the control measure *c*_5_ (i.e., the possible impact of implementing the HBV and HIV treatments control mechanisms) emphasizing a significant reduction of the HBV and HIV co-infected population as compared to a scenario where there is no control mechanism implemented. When comparing the simulation results of the implementation of single control strategies, it has been observed that Strategies 1 (i.e., implementing protection against HBV infection) and 5 (i.e., implementing the treatment strategy for the HBV and HIV co-infection) poses high potential impact to reduce and control the spread of the HBV and HIV co-infection disease spreading in the community.

### Simulations of Scenario B

**Figure 4:**
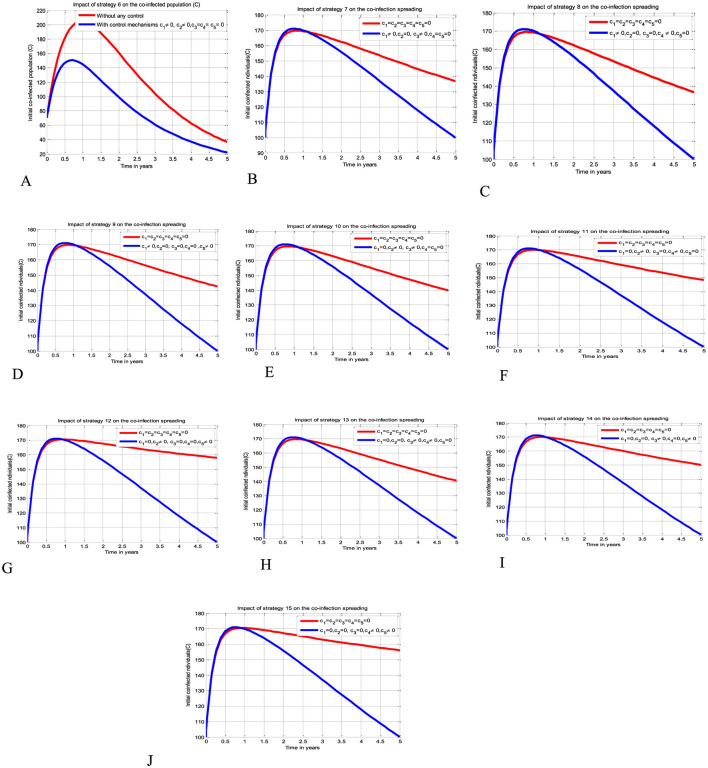
Impacts of double control strategies on the HBV and HIV co-infection spreading. **(A)** Impact of both HBV and HIV protections on the co-infection. **(B)** Impact of HIV protection and HIV treatment on the co-infection. **(C)** Impact of HIV protection and HBV treatment on the co-infection. **(D)** Impact of HIV protection and co-infection treatment on the co-infection. **(E)** Impact of HBV protection and HIV treatment on the co-infection. **(F)** Impact of HBV protection and HBV treatment on the co-infection. **(G)** Impact of HBV protection and co-infection treatment on the co-infection. **(H)** Impact of both HIV and HBV treatments on the co-infection. **(I)** Impact of HIV and co-infection treatment on the co-infection. **(J)** Impact of HBV treatment and co-infection treatment on the co-infection.

The simulation curve illustrated in Figures 4A–J show the possible impacts of implementing double control strategies from Strategy 6-Strategy 15 (i.e., the possible impact of implementing both the HBV and HIV protections control mechanisms *c*_1_ and *c*_2_ simultaneously, the HIV protection and HIV treatment control mechanisms *c*_1_ and *c*_3_ simultaneously, the HIV protection and HBV treatment control mechanisms *c*_1_ and *c*_4_ simultaneously, the HIV protection and co-infection treatment control mechanisms *c*_1_ and *c*_5_ simultaneously, the HBV protection and HIV treatment control mechanisms *c*_2_ and *c*_3_ simultaneously, the HBV protection and HBV treatment control mechanisms *c*_2_ and *c*_4_ simultaneously, the HBV protection and co-infection treatment control mechanisms *c*_2_ and *c*_5_ simultaneously, the HIV treatment and HBV treatment control mechanisms *c*_3_ and *c*_4_ simultaneously, the HIV treatment and co-infection treatment control mechanisms *c*_3_ and *c*_5_ simultaneously, and the HBV treatment and co-infection treatment control mechanisms *c*_4_ and *c*_5_ simultaneously, respectively) emphasizing a significant reduction of the HBV and HIV co-infected population as compared to a scenario implemented in Figures 3A–E and where there is no control mechanism implemented.

### Simulations for Scenario C

**Figure 5:**
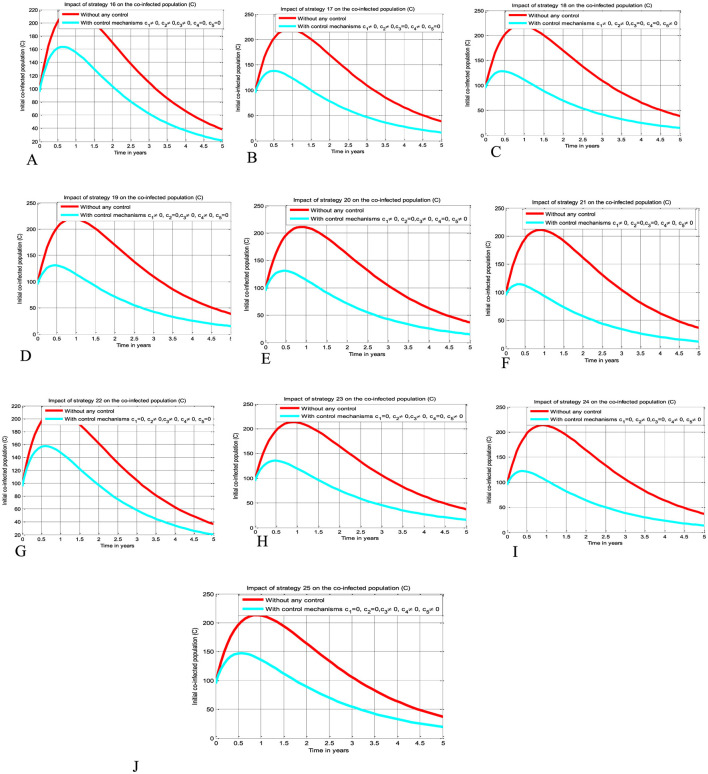
Impacts implementing triple of control Strategies on the HIV and HBV co-infection spreading. **(A)** Impact of Strategy 16 on the co-infection spreading. **(B)** Impact of Strategy 17 on the co-infection spreading. **(C)** Impact of Strategy 18 on the co-infection spreading. **(D)** Impact of Strategy 19 on the co-infection spreading. **(E)** Impact of Strategy 20 on the co-infection spreading. **(F)** Impact of Strategy 21 on the co-infection spreading. **(G)** Impact of Strategy 22 on the co-infection spreading. **(H)** Impact of Strategy 23 on the co-infection spreading. **(I)** Impact of Strategy 24 on the co-infection spreading. **(J)** Impact of Strategy 25 on the co-infection spreading.

The simulation curve illustrated in Figures 5A–J shows the possible impacts of implementing triple control strategies from Strategy 16–Strategy 25 (i.e., the possible impact of implementing *c*_1_≠0, *c*_2_≠0, *c*_3_≠0, *c*_4_ = *c*_5_ = 0 simultaneously, *c*_1_≠0, *c*_2_≠0, *c*_4_≠0, *c*_3_ = *c*_5_ = 0 simultaneously, *c*_1_≠0, *c*_2_≠0, *c*_5_≠0, *c*_3_ = *c*_4_ = 0 simultaneously, *c*_1_≠0, *c*_3_≠0, *c*_4_≠0, *c*_2_ = *c*_5_ = 0 simultaneously, *c*_1_≠0, *c*_3_≠0, *c*_5_≠0, *c*_2_ = *c*_4_ = 0 simultaneously, *c*_1_≠0, *c*_4_≠0, *c*_5_≠0, *c*_1_ = *c*_3_ = 0 simultaneously, *c*_2_≠0, *c*_3_≠0, *c*_4_≠0, *c*_1_ = *c*_5_ = 0 simultaneously, *c*_2_≠0, *c*_3_≠0, *c*_5_≠0, *c*_2_ = *c*_4_ = 0 simultaneously, *c*_2_≠0, *c*_4_≠0, *c*_5_≠0, *c*_1_ = *c*_3_ = 0 simultaneously, and *c*_3_≠0, *c*_4_≠0, *c*_5_≠0, *c*_1_ = *c*_2_ = 0 simultaneously), respectively suggests and emphasizing a significant reduction of the HBV and HIV co-infected population as compared to a scenario implemented in Figures 3A–E, 4A–J, and where there is no control mechanism implemented. By comparing the simulation results of the implementation of each proposed triple control strategies simultaneously, we observed and suggested that Strategies 18, 21, and 24 have more potential to reduce and control the HBV and HIV co-infection disease spreading in the community.

### Simulations for Scenario D

**Figure 6:**
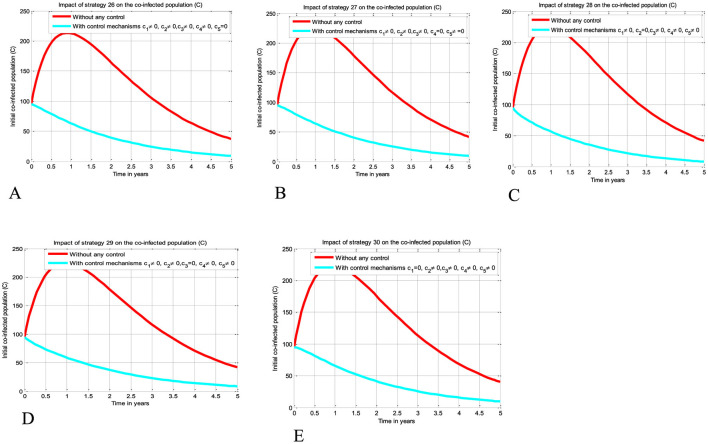
Impact of implementing quadruple strategies simultaneously on the co-infection. **(A)** Impact of Strategy 26 on the co-infection. **(B)** Impact of Strategy 27 on the co-infection. **(C)** Impact of Strategy 28 on the co-infection. **(D)** Impact of Strategy 29 on the co-infection. **(E)** Impact of Strategy 30 on the co-infection.

The simulation curve illustrated in Figures 6A–E shows the impact of implementing quintuple control strategies from Strategy 26 to Strategy 30 (i.e., impact of implementing *c*_1_≠0, *c*_2_≠0, *c*_3_≠0, *c*_4_≠0, *c*_5_ = 0 simultaneously, the HIV protection and HIV treatment control mechanisms *c*_1_ and *c*_3_ simultaneously, *c*_1_≠0, *c*_2_≠0, *c*_3_≠0, *c*_5_≠0, *c*_4_ = 0 simultaneously, *c*_1_≠0, *c*_3_≠0, *c*_4_≠0, *c*_5_≠0, *c*_2_ = 0 simultaneously, *c*_1_≠0, *c*_2_≠0, *c*_4_≠0, *c*_5_≠0, *c*_3_ = 0 simultaneously, and *c*_2_≠0, *c*_3_≠0, *c*_4_≠0, *c*_5_≠0, *c*_1_ = 0 simultaneously, respectively) shows a significant reduction of the HBV and HIV co-infected population as compared to a scenario implemented in Figures 3A–E, 4A–J, 5A–J, and where there is no control mechanism implemented. Comparing the simulation results of the implementation of quintuple control strategies simultaneously we observed that Strategies 1 (i.e., implementing protection against HBV infection) and 30 (i.e., implementing all the treatment strategies and the HBV protection measure) have great potential impact to reduce and control the HBV and HIV co-infection disease spreading in the community.

### Simulations for Scenario E

**Figure 7:**
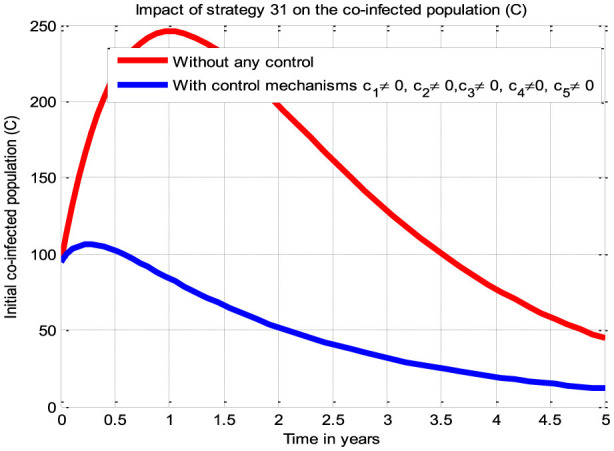
Impact of all the proposed control measures on the co-infection.

The simulation curve illustrated in Figure 7 shows the potential impact of Control Strategy 31 (i.e., the impact of implementing all five proposed control mechanisms simultaneously), emphasizing a significant reduction of the HBV and HIV co-infected populations as compared to the control strategies illustrated in scenarios A, B, C, and D and given in Figures 3–6, respectively, where there is no control mechanism implemented. Therefore, we observed that Strategy 31 has more potential to reduce and control the HBV and HIV co-infection disease spreading in the community.

### Cost-effectiveness analysis of the optimal control problem

In this part, we carried out an analysis of the optimal control strategies cost-effectiveness, investigate, and compare the potential cost benefits for the control strategies that are incorporated in the optimal control problem of the study. The cost-effectiveness analysis of the implemented control strategies is performed by using the same approach outlined in reference (39), namely the incremental cost-effectiveness ratio (ICER). The mathematical formula to compute this result is defined by


$$\begin{array}{l} \text{ICER }=\;\frac{\text{Change in total costs in strategies A and B}}{\text{Change in control benefits in strategies A and B}}, \end{array}$$

where ICER numerator includes the differences in addiction averted costs, costs of protected cases, treatment costs, among others. While the denominator of ICER accounts for the differences in health outcomes, including the total number of infected cases averted or the total number of susceptibility cases protected. The criteria used to evaluate the cost-effectiveness of different infection control measures are analyzed the cost-effectiveness by ranking the control strategies in increasing order of effectiveness in terms of the number of infected averted and leaving the strategy with the dominant ICER value. Therefore, in this section of the study, we performed the cost-effectiveness analysis based on the numerical simulations of the optimal control problem (Equation 12). Thus, similar approach used in several previous studies like (39, 46), the incremental cost-effectiveness ratio (ICER) is calculated to determine the potential cost-effectiveness of all the different control intervention strategies considered in this study. According to the numerical simulation results of the optimal control problem (Equation 12), from the 31 control strategies grouped by five scenarios, namely, Scenario A, Scenario B, Scenario C, Scenario D, and Scenario E, respectively implementing each of the strategies in a given scenario are possibly ranked in ascending order with respect to the total number of infections averted, as shown in Table 4.

**Table 4:** Number of infections averted for Strategies 1–5 in an increasing order.

**Strategy**	**Total number of infections averted**	**Total cost (in USD $)**	**ICER**
Strategy 4	6.0015 × 10^7^	0.041 × 10^3^	6.8163 × 10^−7^
Strategy 3	6.0329 × 10^7^	0.069 × 10^3^	8.9172 × 10^−5^
Strategy 5	6.1242 × 10^7^	0.33 × 10^3^	2.8587 × 10^−4^
Strategy 2	2.0309 × 10^8^	1.12 × 10^3^	5. 5693 × 10^−6^
Strategy 1	2.1712 × 10^8^	0.74 × 10^3^	−2.7084 × 10^−5^

### Cost-effectiveness for Scenario A

Here, ICER is computed for the controlling strategies 4 and 3 in order to compare the two comparative strategies incrementally determined as:


$$\begin{array}{lll} \text{ICER }\left(\text{Strategy }4\right) & = & \frac{0.041\times 10^{3}}{6.0015\times 10^{7}}=6.8163\times 10^{-\;7},\\ \text{ICER }\left(\text{Strategy }3\right) & = & \;\frac{0.069\times 10^{3}-0.041\times 10^{3}}{6.0329\times 10^{7}-6.0015\times 10^{7}}=8.9172\times 10^{-\;5},\\ \text{ICER }\left(\text{Strategy }5\right) & = & \frac{0.33\times 10^{3}-0.069\times 10^{3}}{6.1242\times 10^{7}-6.0329\times 10^{7}}=2.8587\times 10^{-\;4},\\ \text{ICER }\left(\text{Strategy }2\right) & = & \frac{1.12\times 10^{3}-0.33\times 10^{3}}{2.0309\times 10^{8}-6.1242\times 10^{7}}=5.\;5693\times 10^{-\;6},\\ \text{ICER }\left(\text{Strategy }1\right) & = & \frac{0.74\times 10^{3}-1.12\times 10^{3}}{2.1712\times 10^{8}-2.0309\times 10^{8}}=-2.7084\times 10^{-\;5}, \end{array}$$

The analysis in Table 4 revealed that the ICER of Strategy 3 is higher than the ICER of Strategy 4, showing that Strategy 3 is more costly and has lower effectiveness compared with other strategies. Because the strategy was too expensive and less effective, we excluded other alternative strategies that were competing for limited resources, resulting in the re-computed ICER for Strategies 4 and 5.

Based on the data presented in Table 5, we determined that Strategy 5 should be eliminated as its ICER value was higher than that of Strategy 4. Table 6 shows the results of the computation we performed to compare Strategies 4 and 2.

**Table 5:** Comparison of Strategies 4 and 5.

**Strategy**	**Total number of infections averted**	**Total cost (in USD $)**	**ICER**
Strategy 4	6.0015 × 10^7^	0.041 × 10^3^	6.8163 × 10^−7^
Strategy 5	6.1242 × 10^7^	0.33 × 10^3^	2.3635 × 10^−4^
Strategy 2	2.0309 × 10^8^	1.12 × 10^3^	5. 6211 × 10^−6^
Strategy 1	2.1712 × 10^8^	0.74 × 10^3^	−2.7084 × 10^−5^

**Table 6:** Comparison of Strategies 4 and 2.

**Strategy**	**Total number of infections averted**	**Total cost (in USD $)**	**ICER**
Strategy 4	6.0015 × 10^7^	0.041 × 10^3^	−7.5248 × 10^−5^
Strategy 2	2.0309 × 10^8^	1.12 × 10^3^	5. 5693 × 10^−6^
Strategy 1	2.1712 × 10^8^	0.74 × 10^3^	−2.7084 × 10^−5^

We have eliminated Strategy 2 and proceeded with the procedures to compare Strategies 4 and 1, as shown in Table 7. The results shown in Table 6 indicate that Strategy 2 is more cost-effective than Strategy 4.

**Table 7:** Comparison of Strategies 4 and 1.

**Strategy**	**Total Number of infections averted**	**Total cost (in USD $)**	**ICER**
Strategy 4	6.0015 × 10^7^	0.041 × 10^3^	−7.5248 × 10^−5^
Strategy 1	2.1712 × 10^8^	0.74 × 10^3^	4.4493 × 10^−5^

Since Strategy 1 exceeds Strategy 4 in terms of cost, as indicated by the result shown in Table 7, we eliminated Strategies 1 and 4, which have high potential cost-effectiveness in scenario A.

### Cost-effectiveness for Scenario B

After the detailed computations of ICER by using the same criteria applied in Scenario A above for all the ten possible control strategies described in Scenario B at the last step of the computations we obtained the result given in Table 8.

**Table 8:** Comparison of Strategies 15 and 8.

**Strategy**	**Total number of infections averted**	**Total cost (in USD $)**	**ICER**
Strategy 15	6.3354 × 10^8^	0.235 × 10^3^	3.709 × 10^−7^
Strategy 8	7.6120 × 10^8^	0.570 × 10^3^	2.6241 × 10^−6^

From the result given in Table 8, we eliminated Strategy 8 since the ICER (Strategy 8) is bigger than the ICER (Strategy 15), and we found that Strategy 15 has the highest cost-effectiveness of all the proposed control strategies described in scenario B above. After the detailed computations of ICER by using the same criteria applied in Scenario A above for all the ten possible control strategies described in Scenario B, and after the last step of the computations, we obtained the result given in Table 9.

**Table 9:** Comparison of Strategies 24 and 21.

**Strategy**	**Total number of infections averted**	**Total cost (in USD $)**	**ICER**
Strategy 24	4.3354 × 10^8^	0.3110 × 10^3^	7.11735 × 10^−7^
Strategy 21	5.6120 × 10^8^	0.4230 × 10^3^	28.7733 × 10^−7^

From the result given in Table 9 we eliminated Strategy 21 since the ICER (Strategy 21) is bigger than the ICER (Strategy 24), and we found that Strategy 24 is the most cost-effective strategy of all the proposed control strategies described in scenario C above.

### Cost-effectiveness for Scenario D

In Table 10, the ICER is computed for the controlling Strategies 29 and 30 in order to compare the two comparative strategies, and the analysis in Table 10 revealed that the ICER of Strategy 30 is higher than the ICER of Strategy 29, showing that Strategy 30 is more costly and less effective. Because the strategy was too expensive and less effective, we excluded other alternative strategies that were competing for limited resources, resulting in the re-computed ICER for Strategies 29 and 28.

**Table 10:** Number of infections averted for Strategies 26–30 in an increasing order.

**Strategy**	**Total number of infections averted**	**Total cost (in USD $)**	**ICER**
Strategy 29	6.0039 × 10^7^	0.045 × 10^3^	6.8316 × 10^−8^
Strategy 30	6.0329 × 10^7^	0.072 × 10^3^	9.8726 × 10^−6^
Strategy 28	6.1242 × 10^7^	0.45 × 10^3^	4.1402 × 10^−4^
Strategy 27	2.0309 × 10^8^	1.34 × 10^3^	6.2760 × 10^−7^
Strategy 26	2.1712 × 10^8^	1.87 × 10^3^	3.776 × 10^−4^

We employed a similar methodology, and based on the data presented in Table 11, we determined that Strategy 28 should be removed because its ICER value was higher than that of Strategy 29. Table 12 shows the results of the computation we conducted to compare Strategies 29 and 27.

**Table 11:** Comparison of Strategies 29 and 28.

**Strategy**	**Total number of infections averted**	**Total cost (in USD $)**	**ICER**
Strategy 29	6.0039 × 10^7^	0.045 × 10^3^	6.8316 × 10^−8^
Strategy 28	6.1242 × 10^7^	0.45 × 10^3^	4.1402 × 10^−4^
Strategy 27	2.0309 × 10^8^	1.34 × 10^3^	6.2760 × 10^−7^
Strategy 26	2.1712 × 10^8^	0.87 × 10^3^	−3.3499 × 10^−5^

**Table 12:** Comparison of Strategies 29 and 27.

**Strategy**	**Total number of Infections Averted**	**Total cost (in USD $)**	**ICER**
Strategy 29	6.0039 × 10^7^	0.045 × 10^3^	6.8316 × 10^−8^
Strategy 27	2.0309 × 10^8^	1.34 × 10^3^	9.0791 × 10^−6^
Strategy 26	2.1712 × 10^8^	0.87 × 10^3^	−3.3499 × 10^−5^

As shown in Table 12, we have eliminated Strategy 27 and proceeded with the procedures to compare Strategies 29 and 26. Strategy 29 is more cost-effective than Strategy 27. Table 13 shows the outcomes of the computation we conducted to compare Strategies 29 and 26.

**Table 13:** Comparison of Strategies 29 and 26.

**Strategy**	**Total number of infections averted**	**Total cost (in USD $)**	**ICER**
Strategy 29	6.0039 × 10^7^	0.045 × 10^3^	6.8316 × 10^−8^
Strategy 26	2.1712 × 10^8^	0.87 × 10^3^	5.2772 × 10^−6^

Since Strategy 26 exceeds Strategy 28 in terms of cost, as indicated by the result shown in Table 13, we eliminated Strategies 26 and 29 as the most cost-effective strategies in scenario D.

### Cost-effectiveness for Scenario E

From Table 14, it has been observed that Strategy 31 is the only control strategy given in Scenario E, and it is the most cost-effective strategy in Scenario E described above.

**Table 14:** Strategy 31.

**Strategy**	**Total number of infections averted**	**Total cost (in USD $)**	**ICER**
Strategy 31	5.1732 × 10^7^	2.5273 × 10^3^	4.8853 × 10^−6^

In Table 15, we have collected each of the control strategies having high potential from Scenario A, Scenario B, Scenario C, Scenario D, and Scenario E, respectively, described above, and computed the control strategy having great potential as compared with other possible strategies used to reduce and control the HIV and HBV co-infection spreading in the community.

**Table 15:** Comparison between control intervention Strategies 31 and 4.

**Strategy**	**Total number of infections averted**	**Total cost (in USD $)**	**ICER**
Strategy 31	5.1732 × 10^7^	2.5273 × 10^3^	4.8853 × 10^−5^
Strategy 4	6.0015 × 10^7^	0.041 × 10^3^	−3.0013 × 10^−4^
Strategy 29	6.0039 × 10^7^	0.045 × 10^3^	1.6667 × 10^−4^
Strategy 24	4.3354 × 10^8^	0.3110 × 10^3^	7.1218 × 10^−7^
Strategy 15	6.3354 × 10^8^	0.235 × 10^3^	−3.800 × 10^−7^

### Cost-effectiveness for the collected cost-effective strategies from each scenario

From the ICER values computed in Table 15, it is observed that Strategy 31 exceeded the Strategy 4 in terms of cost, as indicated by the result shown in Table 15. This implies that the implementation of Strategy 31 will be more costly and less effective than the implementation of Strategy 4. Hence, Strategy 31 is eliminated from the list of alternative control intervention strategies that are competing for the same limited resources. Next, the ICER is finally recalculated for Strategies 4 and 29, as shown in Table 16.

**Table 16:** Comparison between control intervention Strategies 4 and 29.

**Strategy**	**Total number of infections averted**	**Total cost (in USD $)**	**ICER**
Strategy 4	6.0015 × 10^7^	0.041 × 10^3^	6.8316 × 10^−7^
Strategy 29	6.0039 × 10^7^	0.045 × 10^3^	1.6667 × 10^−4^
Strategy 24	4.3354 × 10^8^	0.3110 × 10^3^	7.1218 × 10^−7^
Strategy 15	6.3354 × 10^8^	0.235 × 10^3^	−3.800 × 10^−7^

From the ICER values computed in Table 16, we observed that Strategy 29 exceeds Strategy 4 in terms of cost, as indicated by the result shown in Table 16. This implies that the implementation of Strategy 29 is more costly and less effective than the implementation of Strategy 4. Hence, Strategy 29 is eliminated from the list of alternative control intervention strategies competing for the same limited resources. Next, the ICER is finally recalculated for Strategies 4 and 24, as shown in Table 17.

**Table 17:** Comparison between control intervention Strategies 4 and 24.

**Strategy**	**Total number of infections averted**	**Total cost (in USD $)**	**ICER**
Strategy 4	6.0015 × 10^7^	0.041 × 10^3^	6.8316 × 10^−7^
Strategy 24	4.3354 × 10^8^	0.3110 × 10^3^	7.2284 × 10^−7^
Strategy 15	6.3354 × 10^8^	0.235 × 10^3^	−3.800 × 10^−7^

From the ICER values computed in Table 17, we observed that Strategy 24 exceeds Strategy 4 in terms of cost, as indicated by the result shown in Table 17. This implies that the implementation of Strategy 24 is more costly and less effective than the implementation of Strategy 4. Hence, Strategy 24 is eliminated from the list of alternative control intervention strategies competing for the same limited resources. Next, the ICER is then finally recalculated for Strategies 4 and 15, as shown in Table 18.

**Table 18:** Comparison between control intervention Strategies 4 and 24.

**Strategy**	**Total number of infections averted**	**Total cost (in USD $)**	**ICER**
Strategy 4	6.0015 × 10^7^	0.041 × 10^3^	6.8316 × 10^−7^
Strategy 15	6.3354 × 10^8^	0.235 × 10^3^	3.3826 × 10^−7^

From the ICER values computed in Table 18, it is observed that Strategy 4 exceeds the Strategy 15 in terms of cost, as evidenced by the result shown in Table 18. This implies that the implementation of Strategy 4 is more costly and less effective than the implementation of Strategy 15. Hence, Strategy 4 has removed from the list of alternative control intervention strategies that are competing for the same limited resources. We observed that Strategy 15 [i.e., implementing HBV treatment and the HIV and HBV co-infection treatment measures (*c*_4_≠0, *c*_5_≠0, *c*_1_ = *c*_2_ = *c*_3_ = 0) simultaneously] has a high potential for cost-effectiveness among all the 30 proposed control strategies that shall be used to reduce and control the HIV and HBV co-infection spreading in the community under consideration.

## Conclusions and future directions

In this study, we formulated and rigorously analyzed a compartmental model of the HBV and HIV co-infection disease spreading with optimal control theory and cost-effectiveness. In the methods section of the study, the qualitative analyses of the HBV and HIV co-infection model were formulated to investigate the positivity and boundedness of the model solutions, the stability and bifurcation analyses of model equilibrium points, the sensitivity analyses of the co-infection model parameters, and the optimal control problem with time-dependent optimal control. The HBV and HIV co-infection model effective reproduction number ($R_{HB}$) of the model was obtained based on the method of the next-generation matrix. The proposed model has two unique equilibrium points: one is the co-infection model disease-free equilibrium point denoted by $E_{BH}^{0}$, and the other is the disease endemic equilibrium point denoted by ${(E}_{BH}^{*}).$ The local asymptotic stability of the disease-free equilibrium point is investigated by applying the Routh-Hurwiz local stability conditions. The global asymptotic stability of the model's equilibrium is examined using Castillo-Chavez and Song conditions whenever${\;R}_{HB}<1$. The possibility of backward bifurcation has been verified using Castillo-Chavez and Song conditions whenever${\;R}_{HB}<1,$ and the result shows the co-infection dynamical system (4) does not exhibit bifurcation in the backward direction. This result suggests that the co-infection disease can be eradicated from the population whenever${\;R}_{HB}<1$. The sensitivity analyses of the co-infection model parameters have been carried out. By proposing five time-dependent controlling strategies and applying Pontryagin's Maximum Principle, we formulated and analyzed an optimal control problem of the co-infection dynamical system (4). In the results and discussion section, we conducted a numerical simulation of the optimal control problem, verified its qualitative properties, and carried out the cost-effectiveness analysis of different combinations of the proposed controlling strategies. For the numerical simulation of the model, we used a well-known and more efficacious numerical scheme known as the classical Runge–Kutta fourth order (RK4) methods. The numerical outcomes are discussed in the results and discussion section. From the findings of the study we can suggest that implementing all the proposed controlling strategies simultaneously has a great potential to reduce and control the HBV and HIV co-infection spreading in the community. However, a cost-effectiveness analysis found that Strategy 15 [i.e., implementing HBV treatment and the HIV and HBV co-infection treatment measures (*c*_4_≠0, *c*_5_≠0, *c*_1_ = *c*_2_ = *c*_3_ = 0 simultaneously)] has the highest potential of cost-effectiveness among all other 30 proposed control strategies to reduce and control the HIV and HBV co-infection spreading in the community under consideration.

Since formulating a mathematical model is not exhaustive by itself, therefore there are several limitations in the proposed HIV and HBV co-infection model construction process. Some of the limitations of this study are: the model did not consider the well-known infection stages of both the HIV and HBV diseases, the HBV vaccination did not consider, and the study did not incorporate real data. Therefore, potential researchers could modify this study by incorporating the stochastic approach, the fractional order approach, the age structure of individuals, the HBV infection stages, and the environmental factors, and validate the model with real data collected from the study area.

## Data Availability

The original contributions presented in the study are included in the article/supplementary material, further inquiries can be directed to the corresponding author.
